# A gas-to-particle conversion mechanism helps to explain atmospheric particle formation through clustering of iodine oxides

**DOI:** 10.1038/s41467-020-18252-8

**Published:** 2020-09-09

**Authors:** Juan Carlos Gómez Martín, Thomas R. Lewis, Mark A. Blitz, John M. C. Plane, Manoj Kumar, Joseph S. Francisco, Alfonso Saiz-Lopez

**Affiliations:** 1 Instituto de Astrofísica de Andalucía, CSIC, 18008 Granada, Spain; 2 Department of Atmospheric Chemistry and Climate, Institute of Physical Chemistry Rocasolano, CSIC, 28006 Madrid, Spain; 3 School of Chemistry, University of Leeds, LS2 9JT Leeds, UK; 4 Department of Earth and Environmental Science and Department of Chemistry, University of Pennsylvania, Philadelphia, PA 19104-6323 USA

**Keywords:** Atmospheric chemistry, Atmospheric chemistry

## Abstract

Emitted from the oceans, iodine-bearing molecules are ubiquitous in the atmosphere and a source of new atmospheric aerosol particles of potentially global significance. However, its inclusion in atmospheric models is hindered by a lack of understanding of the first steps of the photochemical gas-to-particle conversion mechanism. Our laboratory results show that under a high humidity and low HO_x_ regime, the recently proposed nucleating molecule (iodic acid, HOIO_2_) does not form rapidly enough, and gas-to-particle conversion proceeds by clustering of iodine oxides (I_x_O_y_), albeit at slower rates than under dryer conditions. Moreover, we show experimentally that gas-phase HOIO_2_ is not necessary for the formation of HOIO_2_-containing particles. These insights help to explain new particle formation in the relatively dry polar regions and, more generally, provide for the first time a thermochemically feasible molecular mechanism from ocean iodine emissions to atmospheric particles that is currently missing in model calculations of aerosol radiative forcing.

## Abstract (web summary)

“How iodine-bearing molecules contribute to atmospheric aerosol formation is not well understood. Here, the authors provide a new gas-to-particle conversion mechanism and show that clustering of iodine oxides is an essential component of this process while previously proposed iodic acid does not play a large role.”

## Introduction

The impacts of iodine on tropospheric and stratospheric chemistry were anticipated by seminal work published in the 80s and 90s^1,2^. The characterization of global iodine sources^3,4^ and the demonstration of its ubiquitous presence in the marine boundary layer (MBL)^5,6^, the free troposphere^7,8^, and the stratosphere^9^ have enabled an assessment of the important role of iodine in O_3_ depletion in past, present, and future climate scenarios^5,9–15^. Iodine oxide-driven new particle formation was first observed in mid-latitude coastal locations^16–18^. Its high nucleation and growth rates may enable new iodine particles to survive fast scavenging by background aerosol^19^ and to develop into sea mist and fog^20^. Iodine oxide particles (IOPs) have also been observed in the polar MBL^21–23^. Field observations in diverse locations with completely different meteorological and chemical conditions suggest a wider relevance of this phenomenon in the global atmosphere.

Laboratory studies have investigated the effect of different environmental variables on nm-sized IOPs^17,24–26^. For example, both high temperature and humidity slow down IOP growth and reduce IOP number density relative to colder/drier conditions^24,26^. The I_2_O_3_ and I_2_O_4_ molecules have been observed in the laboratory and linked to IOP formation in dry conditions^27^. However, the O/I ratio measured in the particles collected has been shown to be closer to I_2_O_5_^26^. I_2_O_5_ is the anhydride form of iodic acid (HOIO_2_), which is the proposed composition of liquid IOPs in the humid MBL^28^. Recently, field observations of IO_3_^−^ and HIO_3_^−^ containing cluster anions by chemical ionization–atmospheric pressure interface–time-of-flight mass spectrometry (CI-API-ToF-MS) have been reported^23^. These observations, which employ nitrate (NO_3_^−^) and acetate (CH_3_COO^−^) reagent ions for reaction with the analytes, have been interpreted as resulting from atmospheric gas-phase HOIO_2_ and molecular cluster formation via HOIO_2_ addition steps. Well-established gas-phase kinetics and thermochemistry do not indicate any straightforward route to HOIO_2_. The OIO + OH reaction is predicted to yield HOIO_2_^29^, but the concentration of OH in the MBL is far too low to explain IOP formation. It has been proposed that water plays an important role in generating HOIO_2_ and IOPs^23,30^, although a strong anticorrelation between particle production probability and water vapor has been observed in the field^31^. In order to form HOIO_2_, H_2_O must be able to intercept some of the initial products of the reaction between atomic iodine and O_3_, i.e., IO_x_ (I, IO, and OIO) or I_2_O_y_. The bimolecular reactions of IO_x_ with H_2_O are precluded by their large endothermicities, and termolecular reactions form only weakly bound clusters^32^. A reaction of any of these H_2_O…IO_x_ adducts with O_3_ to form oxyacids requires substantial bond rearrangement and barriers are expected. Recent ab initio studies have found a substantial barrier in the potential energy surface (PES) of the I_2_O_5_ + H_2_O ↔ 2HOIO_2_ reaction^30,33^, precluding not only formation of HOIO_2_ but also a nucleation mechanism depending on the formation of I_2_O_5_ from the HOIO_2_ self-reaction^23,30^.

Here, we present results from flow tube kinetic experiments in order to fill the knowledge gap between gas-phase atmospheric iodine molecules and IOPs. Our results show that water reacts very slowly with atomic iodine and iodine oxides, and that forming particulate HOIO_2_ does not require gas-phase HOIO_2_, implying a limited role of oxyacids in IOP formation, which is instead initiated by clustering of I_x_O_y_. Based on our experiments and calculations, an iodine gas-to-particle conversion mechanism is proposed.

## Results

### Pulsed laser photolysis experiments

**Fig. 1:**
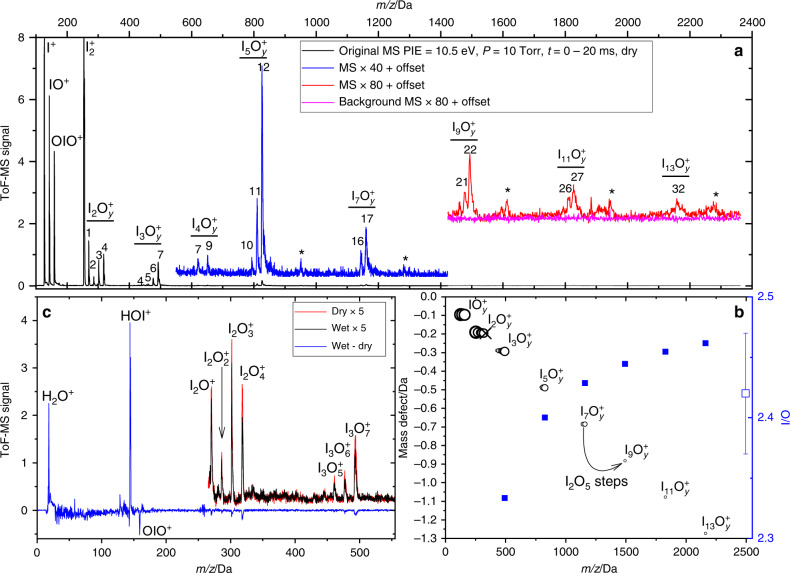
Mass spectra and mass defect of iodine oxides. **a** Time-of-flight mass spectrum (ToF-MS) at photoionization energy (PIE) of 10.5 eV (10 Torr, [O_3_] = 2 × 10^15^ molecule cm^−3^, no added H_2_O) averaged over 20 ms after the photolysis laser pulse. Two sections of the spectrum are displayed with an offset and multiplied by 40 and 100 to show more clearly the peaks corresponding to higher *m/z* clusters. For the third section (red), the corresponding pre-photolysis background is also shown. The asterisks indicate groups of peaks with even number of iodine atoms. ToF-MS signal in arbitrary units. **b** Mass-defect plot (the difference between molecular mass and the integer mass given by the sum of protons and neutrons in the nuclei) for the main clusters observed. The area of the dots is proportional to the corresponding log-scaled signal intensity. Peak progressions in steps of I_2_O_5_ units can be distinguished, but a substantial I_2_O_5_^+^ signal (x symbol) is absent both at 10.5 eV and 11.6 eV. The blue squares indicate the O/I ratio of the main peaks at the right-hand-side axis (uncertainties are smaller than symbol sizes). The empty square with error bar shows the O/I ratio measured elsewhere for iodine oxide particles (IOPs)^26^. **c** In blue, the difference between spectra with and without water (10.5 eV, 4 Torr, [O_3_] = 4 × 10^14^ molecule cm^−3^). Negative peaks indicate losses and positive peaks formation of the corresponding species. Sections of the spectra with and without water are shown (black and red lines, respectively), multiplied by 10 for clarity.

**Fig. 2:**
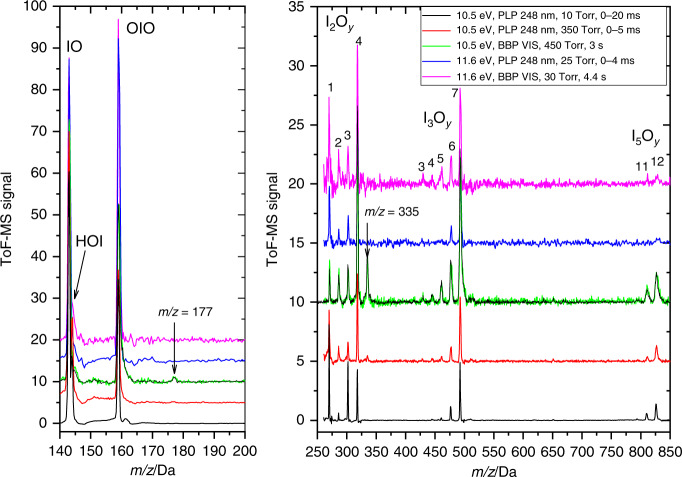
Mass Spectra of iodine oxides in the presence of water vapor. Time-of-flight mass spectra (ToF-MS) acquired under high water mixing ratios (2%) but otherwise different flow tube conditions, photolysis source (PLP pulsed laser photolysis, BBP broadband photolysis), and photoionization energy (PIE). The spectra are displayed in two separated *m/z* ranges for clarity. Except for the changes in the relative differences between the I_2_O_y_ peaks with pressure and the poorer signal-to-noise ratio at  11.6 eV, the spectra look very similar under all conditions studied. The only instances where some new peaks emerge (red and green lines) are for high pressure (350 and 450 Torr). However, these peaks do not depend on the gas-phase water concentration, as illustrated by the 450 Torr BBP dry spectrum (black line superposed to the green line). These peaks correspond to *m/z* = 177 and *m/z* = 355 and were measured at 10.5 eV, i.e., they can neither be assigned to HOIO_2_^+^ nor I_2_O_5_^+^. They were also observed in our previous study under dry conditions^27^.

We have carried out laboratory flow tube kinetic experiments to look at IOP formation and evaporation (see “Methods” and Supplementary Figs. 1 and 2) starting from standard chemistry (Supplementary Table 1). Figure 1a shows an average of mass spectra recorded for time delays of up to 20 ms between 248 nm pulsed laser photolysis (PLP) of a mixture of O_3_ and I_2_ at 10 Torr of N_2,_ and 10.5 eV photoionization (PI). This “dry” spectrum shows positive ion peaks generated by near-threshold PI (Supplementary Table 2) of known neutral iodine species: I^+^ (*m/z* = 127), IO^+^ (143), OIO^+^ (159), I_2_^+^ (254), I_2_O^+^ (270), I_2_O_2_^+^ (286), I_2_O_3_^+^ (302), and I_2_O_4_^+^ (318). A peak corresponding to I_2_O_5_^+^ (*m/z* = 334) is absent. The predicted vertical ionization potential of I_2_O_5_ is 11.4 eV, but this molecule is not observed with the 11.6 eV PI beam either, as shown in Fig. 2. The major peaks of the higher m/z progressions observed in Fig. 1a are I_3_O_7_^+^ (493), I_5_O_12_^+^ (827), I_7_O_17_^+^ (1161), I_9_O_22_^+^ (1495), I_11_O_27_^+^ (1829), and I_13_O_32_^+^ (2163). Minor peaks which indicate up to four missing oxygen atoms are also observed. Starting from I_3_O_7_^+^ and moving to higher m/z, the peaks are separated by I_2_O_5_ units (Fig. 1b), even though gas-phase I_2_O_5_ does not appear to form. The chemical composition of the main and last peak of each progression can be described as (I_2_O_5_)_n_OIO^+^. Molecular clusters with even numbers of iodine atoms (I_4_O_7_^+^, I_4_O_9_^+^) are also detected but are very minor.

**Fig. 3:**
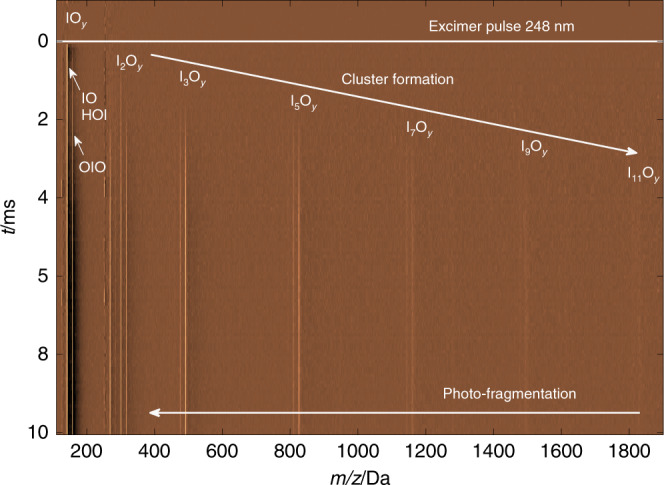
Example of time-resolved mass spectrum. The experiment was started by 248-nm pulsed laser photolysis (PLP) of O_3_ in the presence of I_2_ at 10 Torr with 2% water mixing ratio (6.5 × 10^15^ molecule cm^−3^). The average signal level before PLP has been subtracted. Clear lines indicate time evolution of high signals at certain *m/z* values (color-coded time-of-flight mass spectrometry (ToF-MS) signal in arbitrary units). Formation of IO (*m/z* = 143), HOI (144) and OIO (159) is followed by the appearance of I_2_O_*y*_, I_3_O_*y*_, and larger clusters with the odd number of iodine atoms. At late times all traces grow similarly as a result of photofragmentation of large I_x_O_y_. The time-resolved mass spectra obtained for humid conditions are almost identical to those obtained under dry conditions, except for the presence of HOI and a slight decrease of all I_*x*_O_*y*_ signals. In this experiment, HOIO_2_ would not be expected to be observed due to the photoionization energy (PIE) of 10.5 eV below the ionization threshold.

**Fig. 4:**
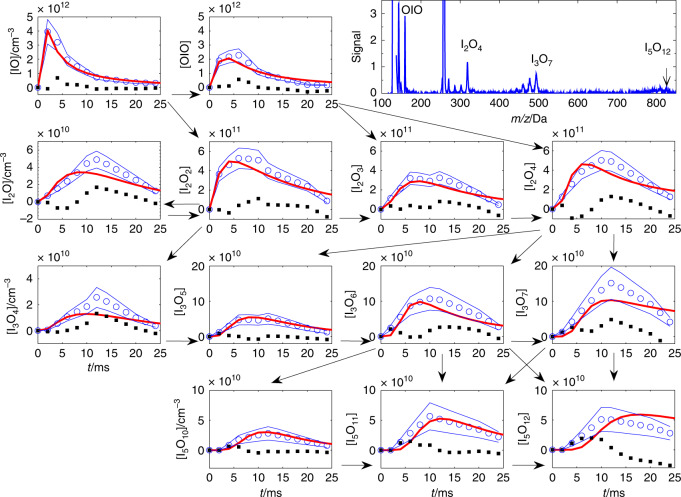
I_*x*_O_*y*_ kinetics. Kinetic traces (blue circles, blue lines estimated statistical uncertainty from signal scatter) of the main peaks (blue line in the right upper panel) observed in a pulsed laser photolysis (PLP) experiment initiated by 193-nm photolysis of I_2_ in the presence of O_3_ at 9 Torr, with 10.5 eV photoionization (PI). The corresponding mass spectrum averaged for all delays is shown in the upper right corner panel. The data were recorded in the digital mode, and the PLP-PI delays were set manually in the delay generator. All traces (in concentration in cm^−3^ versus time in ms) have been interpolated to a constant step time axis. Note that each row shows I_*x*_O_*y*_ with the same number of iodine atoms. The vertical scale is the same for all members of each row, except for the first members of the second and third rows. The red lines show the kinetic traces obtained from a numerical model (Supplementary Tables 1 and 3), and the black squares show the difference between the measurements and the model. The arrows show the main paths assumed in the chemical mechanism (Table 1).

A succession of peaks in a mass spectrum does not necessarily reveal how a nucleation mechanism works. Laboratory time-resolved multiplexed experiments (Fig. 3) provide the sequence of reactions and are therefore essential to interpret mass spectra obtained in the field or in the chamber or flow tube steady-state experiments. The initial growth of the concentration versus time curves is generally faster for smaller iodine-bearing molecules, and the peak concentrations are reached in the following order: IO → OIO → I_2_O_3_ → I_2_O_4_ → I_3_O_7_ → I_5_O_12_ → I_7_O_17_ → I_9_O_22_, etc. (see kinetic analysis in Fig. 4). The kinetic traces indicate that I_2_O_*y*_ form I_3_O_y_ species preferentially over I_4_O_y_ adducts (I_4_O_*y*_ photofragmentation to form I_3_O_*y*_^+^ + IO_*x*_ would occur well above 10.5 eV, Supplementary Table 2). At longer time delays between PLP and PI, all peaks with the number of iodine atoms *x* ≤ 2 show kinetic growth at a similar rate compared with the larger clusters, indicating that the latter fragment into smaller constituent subunits following PI. This growth occurs at earlier delay times when the initial concentration of IO is higher (higher excimer laser energy or higher O_3_ concentration, Supplementary Fig. 3) and/or the PI energy is higher. Because of the second-order reactions involved, starting with the IO self-reaction^34^, the formation and removal time scales of I_*x*_O_*y*_ depend on the initial concentration of IO, which itself depends on the fraction of O_3_ photolyzed. Thus, for high IO, I_*x*_O_*y*_ clustering is fast and a dominant sink compared to other slow reactions.

Addition of water (mixing ratio *x*(H_2_O) = 2%) at 10 Torr results in a limited reduction of the OIO^+^ and I_*x*_O_*y*_^+^ signals (Fig. 1c). No reaction products are detected, except for the peak at *m/z* = 144, i.e., HOI^+^; this results from OH scavenging by I_2_, which also prevents any further HO_*x*_ chemistry. The mass peak progressions in the presence of water remain the same: neither HOIO_2_ nor HOIO_2_-containing peaks (e.g., (I_2_O_5_)_*n*_(HIO_3_)_0–2_^23^) are observed, and no clusters with an even number of iodine atoms emerge. The calculated ionization potential of HOIO_2_ is 11.3 eV, yet again no signal is observed on a time scale of 4 ms when the PI beam is tuned to 11.6 eV (Fig. 2). Regarding larger oxyacid-containing clusters, the PI threshold is expected to decrease as the size of a cluster increases, and therefore they should photoionize or even photofragment at 10.5 eV. Thus, any hypothetical I_x_O_y_ + H_2_O reaction forming HOIO_2_ occurs at a slower rate than the I_*x*_O_*y*_ clustering reactions under these conditions. Given the lack of reaction products upon addition of water, the limited decrease of the I_*x*_O_*y*_ signals indicates that water partially inhibits the formation of large clusters, which results in a reduction of the photofragmentation signal.

Observing slow reactions requires increasing the concentration of the excess reactant (H_2_O) and minimizing the effect of competitive reactions. At 350 Torr and *x*(H_2_O) = 2%, for PLP-PI delays of up to 5 ms, the peaks observed at low pressure in the mass spectrum remain, and oxyacid clusters do not appear (Fig. 2). The minor peaks at *m/z* = 177 (H_2_IO_3_^+^) and *m/z* = 335 (HI_2_O_5_^+^) do not exhibit kinetic change and are decoupled from photolysis-induced chemistry. Thus, they probably result from photofragmentation of large H_2_O…I_*x*_O_*y*_ clusters or are produced from deposits around the ToF-MS skimmer.

### Continuous broadband photolysis (BBP) experiments

We have also performed high-pressure steady-state experiments using continuous BBP. In these experiments, a residence time of a few seconds combined with continuous generation of I atom and high pressure should help in enhancing the concentration of slow-reaction products. Yet again, oxyacid products are not observed under these conditions (Fig. 2). Upper limits for the rate constants of possible HOIO_2_-forming reactions can be obtained from kinetic modeling of our BBP experiments, and are discussed below. Separate flow tube measurements of atomic iodine by resonance fluorescence (ROFLEX)^35^ were performed to study the removal of I atoms in the presence of MBL-representative concentrations of O_3_ and H_2_O at 760 Torr. A slight enhancement of the I atom removal by O_3_ in the presence of water was observed (Supplementary Fig. 4), which yields an effective rate constant of *k*(I + H_2_O + O_3_) = (2.9 ± 1.0) × 10^−19^ cm^3^ s^−1^ molecule^−1^ for *x*(O_3_) = 92 ppbv and *k*(I + H_2_O + O_3_) = (2.0 ± 0.5) × 10^−18^ cm^3^ s^−1^ molecule^−1^ for *x*(O_3_) = 184 ppbv.

**Fig. 5:**
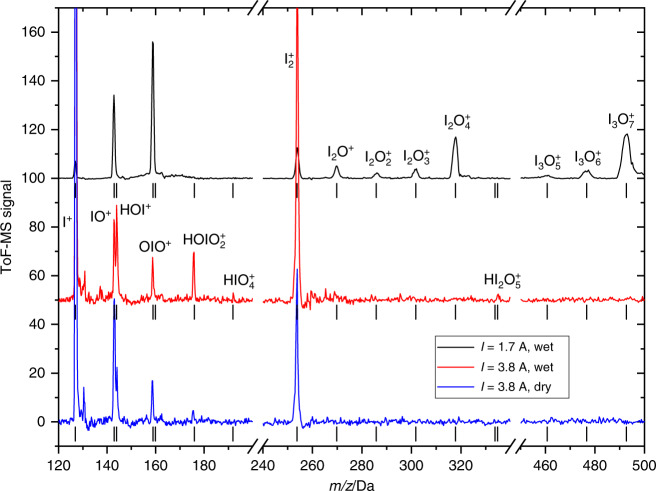
Iodine oxide particles (IOP) pyrolysis experiments at 11.6 eV. The particles are grown along the flow tube, and the carrier gas and particles are passed through a 1-cm diameter resistively heated filament coil just before sampling to detect evaporation products. At a lower filament current of I = 1.7 A (lower temperature at the sampling point), the spectrum obtained at 30 Torr with 2% water mixing ratio resembles other broadband photolysis (BBP) spectra in Fig. 2. When the current through the coil is higher (3.8 A), the spectrum changes (red line) as a result of particle evaporation: the higher masses disappear, the I_2_ signal increases and new masses show up at *m/z* = 144 (HOI^+^) and *m/z* = 176 (HOIO_2_^+^). When water is not added actively to the flow, the oxyacid ion signals decrease drastically, but still some detectable signals remain due to release of water from the inner walls of the experiment. The vertical black lines indicate the position of the oxide and oxyacids in the m/z axis: from left to right: I, IO, HOI, OIO, HOIO, HOIO_2_, HIO_4_, I_2_, I_2_O, I_2_O_2_, I_2_O_3_, I_2_O_4_, I_2_O_5_, HI_2_O_5_, I_3_O_5_, I_3_O_6_, I_3_O_7_. Time-of-flight mass spectrum (ToF-MS) signal in arbitrary units.

Visual inspection of the BBP experiment through a viewport revealed a plume of smoke formed at the I_2_ inlet under dry conditions (Supplementary Movie 1), which was quenched upon addition of H_2_O. That is, water appears to hinder nucleation, rather than promoting it by forming HOIO_2_. The observation of HOIO_2_ evaporated from particles by resistive heating demonstrates the capability of detecting this molecule by PI-ToF-MS at 11.6 eV and provides insights into the formation of particulate HOIO_2_ (Fig. 5). BBP of I_2_/O_3_ mixtures at room temperature generates I_*x*_O_*y*_ and IOPs that travel for a few seconds down the flow tube toward the detection region. A few millimeters upstream of the sampling volume, the carrier gas and the particles are resistively heated (Supplementary Fig. 2) in order to observe evaporation products. The current through the filament was set to a value such that the I_*x*_O_*y*_ molecules carried by the gas thermally dissociated in the absence of water (disappearance of the higher mass peaks). Thus, the observed HOIO_2_ and HOI cannot be products of gas-phase high-temperature I_x_O_y_ + H_2_O reactions, but evaporation products of IOPs formed upstream in the presence of water. Under dry conditions, the oxyacid evaporation signals reduce drastically. The other effect of heating IOPs is the expected thermal decomposition of solid/liquid-phase iodine oxides into molecular oxygen and iodine^36,37^ (enhancement of the I_2_ signal).

### Iodine oxide particle-formation mechanism

The chemistry initiating the formation of iodine oxide clusters and particles is reasonably well known (Supplementary Table 1). Therefore, deviations from the phenomenological growth and decay of IO and OIO documented in previous work^34,38^ are considered as a sign of fragmentation, especially if the IO and OIO traces track the growth of larger molecules and clusters. Data at high IO_x_ concentration clearly suffer from this problem (e.g., experiments #2 and #3 in Supplementary Fig. 3) and cannot be used for kinetic analysis, but it must be pointed out that all datasets are affected to some extent at long delay times. Some of the fragmentation energies of large molecules listed in Supplementary Table 2 are just slightly higher than 10.5 eV or even lower. Two-photon processes may also occur like in the case of water (see “Methods”), although the reason why water is observed by resonance-enhanced multiphoton ionization (REMPI) with an unfocused laser beam is likely related to the high concentrations employed, orders of magnitude higher than the concentrations of I_*x*_O_*y*_.

The I_2_O^+^ signal that is consistently detected in all experiments may originate from an I_2_O parent neutral rather than from fragmentation, possibly from a bimolecular reaction between IO and the products of its self-reaction. The observation of I_2_O_2_^+^ is consistent with IOIO being one of the major products of the IO self-reaction^34,38^, although the signal is very low even in the high-pressure experiments, which may indicate some fragmentation to IO^+^, or a small PI cross section. I_2_O_3_^+^ and I_2_O_4_^+^ can be linked to the corresponding neutrals, which are known products of the IO + OIO and OIO + OIO reactions. The main cluster progressions observed are separated by Δ(*m/z*) = 334 (I_2_O_5_), while I_2_O_5_ itself does not appear to form. Thus, I_2_O_2_, I_2_O_3_, and I_2_O_4_ are considered to be the precursors to iodine oxide molecular clusters. However, they do not appear to aggregate stepwise to form clusters. The ion clusters detected have the specific elemental composition I_2*n*+1_O_5*n*+*m*_^+^ (*n* ≥ 1, *m* = 0, 1, 2), i.e., odd numbers of iodine atoms. By looking at Supplementary Table 2, it can be seen that while I_4_O_*y*_ species may fragment to smaller ions, they cannot fragment to I_3_O_*y*_^+^, and therefore we assume that the latter corresponds to I_3_O_*y*_ molecules formed in the flow tube.

There are many possible reactions that may generate the observed peaks in the PI mass spectra. Thermochemical data are required to eliminate irrelevant reactions from the chemical mechanism (Table 1). Reactions deemed to be endothermic or forming weakly bound adducts based on our calculations are excluded. For example, I_2_O_*y*_ form generally weak adducts with themselves, and this may explain why the I_4_O_*y*_ signals are negligible and, as a consequence, clusters with even numbers of iodine atoms do not form^32^. The most stable predicted adduct is I_4_O_8_, formed from the I_2_O_4_ self-reaction. However, our ab initio calculations suggest that this reaction also has an exothermic bimolecular channel making I_3_O_7_. Alternative routes to I_3_O_*y*_ are I_2_O_*y*_ + IO_x_ (*y* ≠ 3) followed by ozone-oxidation steps. Ozone has been found not to be required to form IOPs as long as an alternative source of oxygen atoms is available, but its presence leads to enhanced particle formation^26^. Thus, it could help in increasing the O/I ratio within each peak progression if the peaks with lower O/I form first from I_2_O_*y*_ + IO_*x*_.

**Table 1:** Reaction enthalpies and barriers (in brackets) in kJ mol^−1^ of reactions forming iodine oxides and oxyacids.

Reaction	Δ*H*_r_ (298 K), this work^a^	Δ*H*_r_ (298 K), literature^b^
IO + IOIO → I_2_O + OIO	−50	−52
IO + I_2_O_3_ → I_3_O_4_		−14
IO + I_2_O_4_ → I_3_O_5_		−63
OIO + IOIO → I_2_O_3_ + IO	−51	−102, −*66*
OIO + I_2_O_3_ → I_3_O_5_		−16
OIO + I_2_O_3_ → I_2_O_4_ + IO	55	60
OIO + I_2_O_4_ → I_3_O_6_^d^	−83	−74
IOIO + IOIO → I_2_O_3_ + I_2_O	−100	−154
IOIO + I_2_O_3_ → I_2_O_4_ + I_2_O	6	40
I_2_O_3_ + I_2_O_3_ → I_3_O_6_ + I	99	
I_2_O_3_ + I_2_O_4_ → I_3_O_6_ + IO	43	
I_2_O_3_ + I_2_O_4_ → I_3_O_7_ + I	52	
I_2_O_4_ + I_2_O_4_ → I_3_O_6_ + OIO	−12	
I_2_O_4_ + I_2_O_4_ → I_3_O_7_ + IO^c^	−4	
I_2_O + O_3_ → IOIO + O_2_	−137	
I_2_O_2_ + O_3_ → I_2_O_3_ + O_2_	−236	−*220*
I_2_O_3_ + O_3_ → I_2_O_4_ + O_2_	−130	−*95*
I_3_O_6_ + O_3_ → I_3_O_7_ + O_2_	−177	
I + H_2_O → I…H_2_O		−20
I + H_2_O + O_3_ → HOIO_2_ + OH	−83	
I + H_2_O + O_3_ → HOIO + HO_2_	−60	
IO + H_2_O → IO…H_2_O		−16
IO + H_2_O + O_3_ → HOIO_2_ + HO_2_	−112	
OIO + H_2_O → OIO…H_2_O		−27
OIO + H_2_O → HOIO + OH		**134**
IOIO + H_2_O → HOI + HOIO	−11	
I_2_O_3_ + H_2_O → I_2_O_3_…H_2_O	−38	−36
I_2_O_3_ + H_2_O → HOI + HOIO_2_	1 [32]	
I_2_O_4_ + H_2_O → I_2_O_4_…H_2_O		−53, −28
I_2_O_4_ + H_2_O → HOIO…HOIO_2_		−104
I_2_O_4_ + H_2_O → HOIO + HOIO_2_		2.9 [3.8]
I_2_O_5_ + H_2_O → 2HOIO_2_		5 [**40**], −8 [13]
HOIO + HOIO → HOI + HOIO_2_	108 [12]	
HOIO + HOIO → H_2_O + I_2_O_3_	116	
HOIO + HOIO_2_ → HOIO…HOIO_2_		−87
HOIO_2_ + HOIO_2_ → HOIO_2_…HOIO_2_		−107

^a^Normal typescript: B3LYP/6-311 + G(2d,p)(AE). Underlined: CCSD(T)//M06-2X/aug-cc-pVTZ+LANL2DZ.

^b^Calculated from published values of ab initio formation or reaction enthalpies. Normal typescript: CCSD(T)//MP2/aug-cc-pVTZ (AREP) from Galvez et al.^32^; italics: CCSD(T)//B3LYP/aug-cc-pVTZ + AE from Kaltsoyannis and Plane^60^; underlined: CCSD(T)//M06-2X/aug-cc-pVTZ+LANL2DZ from Kumar et al.^30^ (concerted pathways); bold: CCSD(T)/CBS//MP2/aug-cc-pVTZ (ECP28) from Khanniche et al.^33^.

I_2_O_3_, in particular, does not form stable adducts with other IO_*x*_ and I_2_O_*y*_ (including itself)^32^, and we have not found any exothermic bimolecular reaction between I_2_O_3_ and IO_*x*_ or I_2_O_*y*_ (Table 1). However, I_2_O_3_ does not accumulate in the flow tube, as shown in Fig. 4 and Supplementary Fig. 3. Therefore, we assume that it undergoes clustering with larger I_*x*_O_*y*_. Further ab initio calculations are required to investigate if this is the actual fate of I_2_O_3_.

The list of reactions added to the initial chemistry in Supplementary Table 1 are listed in Table 2. The rate constants of the selected reactions are then adjusted to model the fast formation and decay of the I_x_O_y_ species, as shown in Fig. 4 (see the section “Kinetic Analysis” in “Methods”). Some reactions are grouped and assumed to have the same rate coefficient. Some rate coefficients are floated by the least-squares algorithm, while some others are changed manually. Not all coefficients can be allowed to float at the same time. Finding a reasonable result implies a few iterations. The criterion is always to minimize the difference between the measured traces and the simulations.

**Table 2:** Tentative mechanism of iodine oxide cluster formation (initiated by the reactions included in Supplementary Table 1)^a^.

#	Reaction^b^	*k*/cm^3^ s^−1^ molecule^−1c^	Grouped reactions	Fit
1	IO + IOIO → I_2_O + OIO	5 × 10^−12^		Floated
2	OIO + IOIO → I_2_O_3_ + IO	1 × 10^−11^		Floated
3	IOIO + IOIO → I_2_O_3_ + I_2_O	1 × 10^−11^	=2	
4	I_2_O + O_3_ → IOIO + O_2_	8 × 10^−14^		Manual
5	I_2_O_2_ + O_3_ → I_2_O_3_ + O_2_	4 × 10^−14^		Manual
6	I_2_O_3_ + O_3_ → I_2_O_4_ + O_2_	8 × 10^−14^		Manual
7	OIO + IOIO → I_3_O_4_	3 × 10^−12^		Floated
8	IO + I_2_O_4_ → I_3_O_5_	3 × 10^−11^		Floated
9	OIO + I_2_O_4_ → I_3_O_6_	5 × 10^−11^		Floated
10	I_2_O_4_ + I_2_O_4_ → I_3_O_6_ + OIO	5 × 10^−12^		Floated
11	I_2_O_4_ + I_2_O_4_ → I_3_O_7_ + IO	1 × 10^−10^		Floated
12	I_3_O_4_ + O_3_ → I_3_O_5_ + O_2_	8 × 10^−14^		Manual
13	I_3_O_5_ + O_3_ → I_3_O_6_ + O_2_	1 × 10^−13^		Manual
14	I_3_O_5_ + O_3_ → I_3_O_7_ + O_2_	1 × 10^−13^		Manual
15	I_3_O_6_ + I_2_O_3_ → I_5_O_9_	1.7 × 10^−10^		Floated
16	I_3_O_7_ + I_2_O_3_ → I_5_O_10_	1.7 × 10^−10^	=15	
17	I_3_O_5_ + I_2_O_4_ → I_5_O_9_	1.7 × 10^−10^	=15	
18	I_3_O_6_ + I_2_O_4_ → I_5_O_10_	1.7 × 10^−10^	=15	
19	I_3_O_7_ + I_2_O_4_ → I_5_O_11_	1.9 × 10^−10^		Floated
20	I_3_O_6_ + I_3_O_6_ → I_5_O_11_ + IO	4 × 10^−10^		Floated
21	I_3_O_6_ + I_3_O_7_ → I_5_O_11_ + OIO	4 × 10^−10^	=21	
22	I_3_O_6_ + I_3_O_7_ → I_5_O_12_ + IO	2 × 10^−10^		Floated
23	I_3_O_7_ + I_3_O_7_ → I_5_O_12_ + OIO	2 × 10^−10^	=22	
24	I_5_O_9_ + O_3_ → I_5_O_10_ + O_2_	3 × 10^−13^		Manual
25	I_5_O_10_ + O_3_ → I_5_O_11_ + O_2_	3 × 10^−13^		Manual
26	I_5_O_11_ + O_3_ → I_5_O_12_ + O_2_	2 × 10^−13^		Manual
27	I_5_O_9_ + I_2_O_3_ → I_7_O_12_	1.7 × 10^−10^	=15	
28	I_5_O_10_ + I_2_O_3_ → I_7_O_13_	1.7 × 10^−10^	=15	
29	I_5_O_11_ + I_2_O_3_ → I_7_O_14_	1.7 × 10^−10^	=15	
30	I_5_O_12_ + I_2_O_3_ → I_7_O_15_	1.7 × 10^−10^	=15	
31	I_5_O_9_ + I_2_O_4_ → I_7_O_13_	1.9 × 10^−10^	=19	
32	I_5_O_10_ + I_2_O_4_ → I_7_O_14_	1.9 × 10^−10^	=19	
33	I_5_O_11_ + I_2_O_4_ → I_7_O_15_	1.9 × 10^−10^	=19	
34	I_5_O_12_ + I_2_O_4_ → I_7_O_16_	1.9 × 10^−10^	=19	
35	I_5_O_12_ + I_3_O_6_ → I_7_O_17_ + IO	1 × 10^−10^		Floated
36	I_5_O_12_ + I_3_O_7_ → I_7_O_17_ + OIO	3 × 10^−10^		Floated
37	I_5_O_11_ + I_5_O_11_ → IOP	6 × 10^−10^		Floated
38	I_5_O_11_ + I_5_O_12_ → IOP	6 × 10^−10^	=38	
39	I_5_O_12_ + I_5_O_12_ → IOP	7 × 10^−10^		Floated

^a^Ab initio thermochemistry is available up to I_4_O_*y*_. Endothermic reactions and reactions forming weakly bound complexes are not included (see Table 1). For larger clusters, reactions are included in order to explain observed peaks and relative peak intensities.

^b^For simplicity, only the forward association reactions are considered, i.e., unimolecular decomposition of the adduct is ignored, and therefore these are effective rate constants.

^c^Rate constants from the global fit in Fig. 2.

The rapid kinetics and smoke formation are astonishing, considering that the pressure is relatively low and that all reactions are second order. Removal rates of ~50 s^−1^ with concentrations of 10^11^ molecule cm^−3^ (Fig. 4 and Supplementary Fig. 3) involve rate constants of the order of 5 × 10^−10^ cm^3^ s^−1^ molecule^−1^. These are likely to be enabled by the large dipole moments characteristic of iodine oxides^32^ inducing long-range capture forces. Kinetic modeling efforts are rendered semi-quantitative at this point by the unknown contribution of fragmentation of large clusters to the smaller molecules. However, a reasonable global fit to all available traces (Fig. 4) may be obtained using standard chemistry (Supplementary Table 1) and the combination of clustering and bimolecular reactions listed in Table 2. In this I_*x*_O_*y*_ nucleation mechanism, rapid pressure-independent bimolecular reactions between I_*x*_O_*y*_ molecular clusters form larger clusters with the observed odd numbers of iodine atoms ((I_2_O_5_)_*n*_OIO), e.g.:

1 $${\mathrm{I}}_{\mathrm{2}}{\mathrm{O}}_{r} + {\mathrm{I}}_{\mathrm{2}}{\mathrm{O}}_{s} \to {\mathrm{I}}_{\mathrm{3}}{\mathrm{O}}_{t} + {\mathrm{IO}}_{{r} + {s} - {t}}\left(r,\,{s} = {\mathrm{2}} - {\mathrm{4}};\;{t} = {\mathrm{4}} - {\mathrm{7}}\right)$$

2 $${\mathrm{I}}_3{\mathrm{O}}_{u} + {\mathrm{I}}_3{\mathrm{O}}_{v} \to {\mathrm{I}}_{\mathrm{5}}{\mathrm{O}}_{w} + {\mathrm{IO}}_{{u + v - w}}({u},{v} = 4 - 7;{w} = 10 - 12).$$

In addition, third-body reactions at their high-pressure limit form aggregates, e.g.:

3 $${\mathrm{IO}}_{x} + {\mathrm{I}}_{\mathrm{2}}{\mathrm{O}}_{y} \to {\mathrm{I}}_{\mathrm{3}}{\mathrm{O}}_{{x} + {y}}({x} = {\mathrm{1,}}\,{\mathrm{2}};\;{y} = {\mathrm{3}},\,{\mathrm{4}})$$

4 $${\mathrm{I}}_{\mathrm{2}}{\mathrm{O}}_{y} + {\mathrm{I}}_{\mathrm{3}}{\mathrm{O}}_{z} \to {\mathrm{I}}_{\mathrm{5}}{\mathrm{O}}_{{y} + {z}}({y} = {\mathrm{2}} - {\mathrm{4}};\;{z} = {\mathrm{4}} - {\mathrm{7}}),$$

where the O/I ratio of the aggregates may be subsequently increased through oxidation by O_3_. HOIO_2_-containing clusters^23^ have not been observed, and therefore no attempt has been made to model them.

### Constraints to iodic acid-formation rates

Possible sources of gas-phase HOIO and HOIO_2_ are reactions of IO_*x*_ and I_2_O_*y*_ with water listed in Table 1. We have carried out high-level ab initio calculations to explore the transition states toward oxyacid formation from I_2_O_*y*_ + H_2_O (*y* = 2, 3). The gas-phase I_2_O_2_ reaction (Supplementary Fig. 5) is more favorable than the I_2_O_3_ reaction (Supplementary Fig. 6), while the I_2_O_4_ reaction^30^ presents a small barrier (Supplementary Fig. 7). We have also hypothesized that wall reactions of iodine oxides with adsorbed H_2_O may be an additional source of oxyacids in long-residence time experiments and field instruments with long inlets. However, Born–Oppenheimer molecular simulations (BOMD) rule out I_2_O_*y*_ reactions in the air-water interface (see “Methods”).

We have selected five exothermic reactions producing oxyacids listed in Table 1 in order to determine their rate constants from the experimental data and from our ab initio calculations. Active addition of high water concentrations in our BBP experiments at a PI energy of 11.6 eV does not result in a detectable signal of HOIO_2_. Moreover, the co-products of these reactions HOI and HOIO are very weak or absent from the BBP mass spectra obtained at 10.5 eV. This suggests that these reactions are slow and cannot compete under our experimental conditions with the reactions removing I_*x*_O_*y*_ in the “dry” experiments. This, however, does not rule out the possibility that under atmospheric conditions, the situation reverses as a result of the low ambient IO_*x*_ ([IO_*x*_] <10^9^ cm^−3^ have been measured in the field, i.e., three to four orders of magnitude lower than in our BBP experiments) and higher water concentrations. Thus, upper limits to the rate constants of these reactions need to be determined as a first step to establish the relative importance of the oxide and oxyacid clusters. This has been done by kinetic modeling of our long-residence time BBP steady-state experiments at 10.5 eV and 11.6 eV PI energy, where each process in Table 3 is included separately in the numerical model with different values of the rate constant. The calculated concentrations of the oxyacid co-products after a time equal to the residence time in the flow tube are used to scale their simulated peaks, which have the typical mass resolution of the measured spectra in the *m/z* = 140–200 Da range. It is assumed that the PI cross section of each iodine oxyacid is equal to that of the nearest oxide. The upper limit of each rate constant k(I_*x*_O_*y*_ + H_2_O) is taken to be that for which the simulated mass spectrometric signal at *m/z* = 144, *m/z* = 160, and/or *m/z* = 176 becomes indistinguishable from the background noise of the BBP experimental data. These upper limits can then be compared with rate constants calculated with the master equation solver MESMER^39^ using the molecular parameters (energies, vibrational frequencies, and rotational constants) derived from our ab initio calculations (Table 3).

**Table 3:** Calculated rate constants of possible oxyacid sources (*T* = 295 K).

Reaction	*k* MESMER		*k* upper-limit experimental^e^
	30 Torr	760 Torr	
I + H_2_O → H_2_O…I	2.4 × 10^−13^ cm^3^ s^−1^	4.7 × 10^−12^ cm^3^ s^−1^	
H_2_O…I → I + H_2_O	1.7 × 10^6^ s^−1^	3.4 × 10^7^ s^−1^	
H_2_O…I + O_3_ → HOIO/HOIO_2_ + HO_2_/OH	100 s^−1^	100 s^−1a^	
I + H_2_O ( + O_3_)→ HOIO/HOIO_2_ + HO_2_/OH	2.8 × 10^−19^ cm^3^ s^−1^	2.1 × 10^−19^ cm^3^ s^−1^	2.8 × 10^−19^ cm^3^ s^−1^ (**a**, **e**)
IO + H_2_O → H_2_O…IO	1.9 × 10^−15^ cm^3^ s^−1^	4.6 × 10^−14^ cm^3^ s^−1^	
H_2_O…IO → IO + H_2_O	1.6 × 10^7^ s^−1^	4.0 × 10^8^ s^−1^	
H_2_O…IO + O_3_ → HOIO_2_ + HO_2_	100 s^−1^	100 s^−1b^	
IO + H_2_O ( + O_3_) → HOIO_2_ + HO_2_	2.0 × 10^−21^ cm^3^ s^−1^	2.0 × 10^−21^ cm^3^ s^−1^	2.0 × 10^−20^ cm^3^ s^−1^
I_2_O_2_ + H_2_O → HOI + HOIO			1 × 10^−19^ cm^3^ s^−1^ (**b**)
I_2_O_3_ + H_2_O → HOI + HOIO_2_	2.3 × 10^−20^ cm^3^ s^−1^		2 × 10^−18^ cm^3^ s^−1^ (**c**)
	Eckart tunneling^c^: 3.8 × 10^−19^ cm^3^ s^−1^		
I_2_O_4_ + H_2_O → HOIO + HOIO_2_	9.3 × 10^−16^ cm^3^ s^−1^	8.0 × 10^−16^ cm^3^ s^−1^	5 × 10^−19^ cm^3^ s^−1^ (**d**)
→ HOIO…HOIO_2_^d^	0.2 × 10^−16^ cm^3^ s^−1^	1.4 × 10^−16^ cm^3^ s^−1^	

^a^Adjusted to obtain a net rate constant equal to the effective rate constant determined by resonance fluorescence with the ROFLEX machine. For [O_3_] = 2.5 × 10^12^ molecule cm^−3^ (100 ppbv at 760 Torr), the corresponding rate constant would need to be: *k* = 4 × 10^−11^ cm^3^ s^−1^ molecule^−1^.

^b^The same loss rate of the I-water adduct is assumed for the IO-water adduct.

^c^Quantum-mechanical tunneling is incorporated in the master equation using a parabolic Eckart-type barrier^39,61^.

^d^The HOIO…HOIO_2_ potential well is deep enough to enable collisional stabilization of the adduct at high pressure. We have no evidence of a peak at *m/z* = 336 in our high-pressure mass spectra.

^e^Bold letters in brackets refer to the panel of Fig. 6 in the main text showing the simulation from which the upper limit is obtained. For I_2_O_*y*_ + H_2_O, the upper limit is better constrained by the experiment at 450 Torr with 10.5 eV PI photon energy (thee co-products HOI and HOIO have ionization energies below 10.5 eV).

**Fig. 6:**
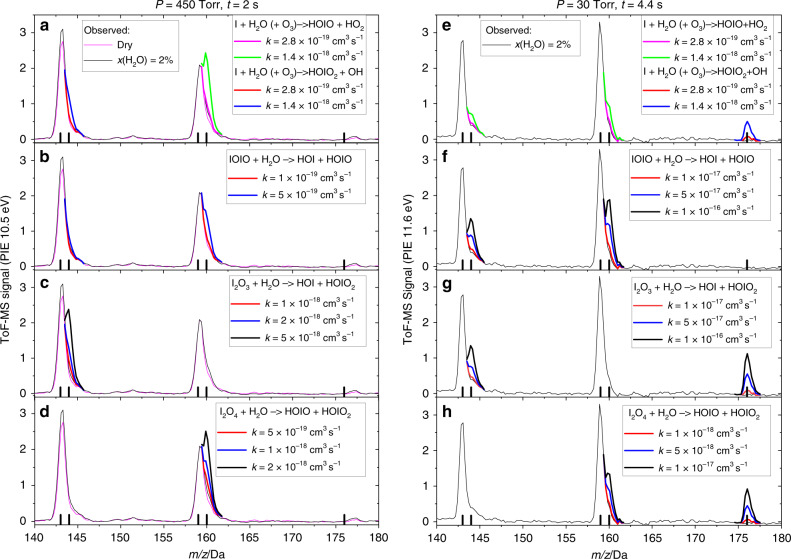
Determination of upper limits to oxyacid-formation rate constants. Time-of-flight mass spectra (ToF-MS) obtained with photoionization energies (PIE) of 10.5 eV (panels **a**–**d**) and 11.6 eV (panels **e**–**h**) in broadband photolysis (BBP) experiments at 450 Torr and 30 Torr, respectively, without added water (blue line) and with 2% water mixing ratio (black line). Note that the labels of *y* and *x* axes are the same for all panels in the same column. The vertical black lines indicate the position of the oxide and oxyacids in the *m/z* axis: from left to right: IO, HOI, OIO, HOIO, HOIO_2_. Simulated oxyacid ion peaks (thick lines) resulting from four different kinetic modeling scenarios with different rate constants are overlaid to the measured spectra: I + H_2_O + O_3_ → HOIO/HOIO_2_ + HO_2_/OH (**a**, **b**) IOIO + H_2_O → HOIO_2_ + HO_2_ (**b**, **f**), I_2_O_3_ + H_2_O → HOI + HOIO_2_ (**c**, **g**), I_2_O_4_ + H_2_O → HOIO + HOIO_2_ (**d**, **h**), and I + H_2_O + O_3_ → OH + HOIO_2_. Note that reactions producing HO_x_ also would result in the generation of HOI (panels **a**, **e**).

A comparison between the simulated ion peaks and the observed mass spectra is shown in Fig. 6. Even though HOIO_2_ is not expected to be observable at 10.5 eV, the data obtained at this photon energy help in reducing the upper limit of the rate constant of the I_2_O_2_ and I_2_O_3_ reactions. First, the concentrations of these two oxides, in particular, are enhanced at high pressure (the IO + OIO and OIO + OIO rate constants reach their high-pressure limit)^34^. Second, the water concentration is one order of magnitude higher as a result of the higher pressure (the S/N ratio at 10.6 eV was not high enough to perform experiments above 30 Torr). The upper limits are listed in Table 3. For the I_2_O_2_ + H_2_O and I_2_O_4_ + H_2_O reactions, the rate constant cannot exceed, respectively, 1 × 10^−19^ cm^3^ s^−1^ molecule^−1^ and 5 × 10^−19^ cm^3^ s^−1^ molecule^−1^. The upper limit of k(I_2_O_3_ + H_2_O) is 5 × 10^−18^ cm^3^ s^−1^ molecule^−1^, but owing to the large barrier of the I_2_O_3_ + H_2_O reaction (Supplementary Fig. 6), its rate constant is expected to be at least one order of magnitude lower, as indicated by the master equation calculation included in Table 3. The I_2_O_2_ + H_2_O reaction and the I_2_O_4_ + H_2_O reaction (concerted path^30^) present, respectively, a submerged barrier of −7.5 kJ mol^−1^ (Supplementary Fig. 5) and a low barrier of 3.4 kJ mol^−1^ (Supplementary Fig. 7) at the CCSD(T)//M06-2X/aug-cc-pVDZ+LANL2DZ level of theory, which yields calculated rate constants higher than the estimated upper limits deduced from the experimental data.

The upper limit determined for the rate constant of a hypothetical composite reaction where atomic iodine complexes with water, and the resulting H_2_O…I adduct reacts with O_3_ to form oxyacids (Fig. 6a, e) can be compared with the rate constants obtained from the resonance fluorescence experiments on the removal of atomic iodine by O_3_ in the presence of water vapor. The enhanced removal of I by O_3_ in the presence of H_2_O observed with the ROFLEX for *x*(O_3_) = 92 ppbv (*k*(I + H_2_O + O_3_) = (2.9 ± 1.0) × 10^−19^ cm^3^ s^−1^ molecule^−1^) appears to agree with the upper limit determined from the ToF-MS experiments: *k*(I + H_2_O + O_3_) < 2.8 × 10^−19^ cm^3^ s^−1^ molecule^−1^. However, this is coincidental. The O_3_ concentration in the ToF-MS experiments was three orders of magnitude larger than in the ROFLEX experiments, indicating that the rate constant in the former should be, if linearly extrapolated from the ROFLEX result, of the order of 2.9 × 10^−16^ cm^3^ s^−1^ molecule^−1^, i.e., well above the detection limit of ~10^−18^ cm^3^ s^−1^ molecule^−1^. Thus, the yield of HOIO and HOIO_2_ from this process is <0.001, implying an oxyacid-formation rate constant of the order of 10^−22^ cm^3^ s^−1^ molecule^−1^ for atmospherically relevant O_3_ concentrations. Therefore, the composite I + H_2_O + O_3_ reaction is not a sufficiently fast source of HOIO_2_. This is very important because, as discussed in Supplementary Note 1, oxyacids can only be effective IOP nucleating species if their formation is decoupled from I_*x*_O_*y*_: otherwise, oxyacid nucleation needs to proceed through two slow second-order processes to form the first cluster. To explore the relative contribution of iodine oxides and oxyacids to the nucleation of IOPs, we have modeled a BBP flow tube experiment with longer residence time (200 s). Calculated rate constants of oxyacid–oxyacid and oxide–oxyacid aggregation and dissociation rates are listed in Supplementary Table 4. Modeled concentrations are presented in Supplementary Fig. 8 as a function of water vapor concentration ([H_2_O] concentrations between laboratory experiments^23^ and ambient). Despite the many assumptions involved in this exercise, a conclusion that can be drawn is that molecular clusters descending directly from HOIO and HOIO_2_ can only become dominant at high water concentration (>5 × 10^16^ molecule cm^−3^), and if the direct precursor of these oxyacids is atomic iodine. If the precursors of these oxyacids are oxides—which is more likely—nucleation is started by iodine oxides.

## Discussion

**Fig. 7:**
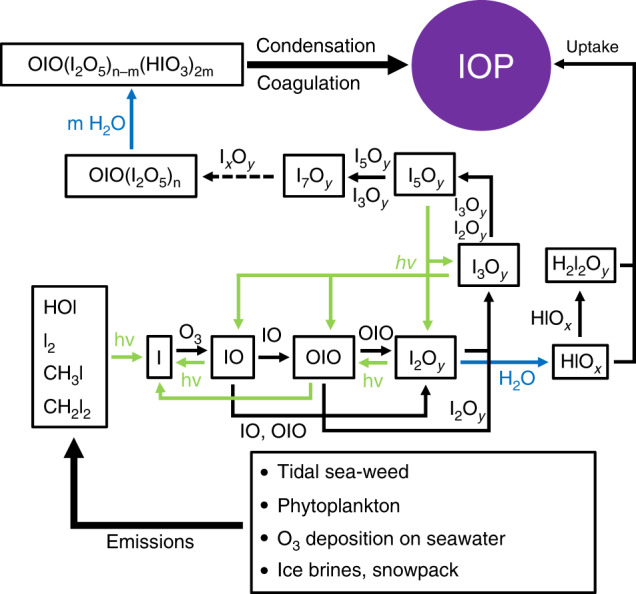
Proposed mechanism of iodine oxide new particle formation. Iodine-bearing molecules are emitted from the ocean surface and marine biota^4,5,64,65^ and photo-oxidized in the presence of O_3_, leading to the formation of OIO^34^ and I_2_O_*y*_ molecules^27^. According to the findings of this work, I_2_O_*y*_ reactions lead to the formation of molecular clusters with composition OIO(I_2_O_5_)_*n*_, which transition into HOIO_2_-containing clusters and iodine oxide particles (IOP) by progressive hydration (the derivation of the mechanism is explained in the “Results” section). Further uptake of water, organic acids, and H_2_SO_4_^26^ leads to particle formation. The formation of iodic acid (HOIO_2_) by the reaction of H_2_O with I_2_O_y_ cannot be disproved, but appears to be too slow for HOIO_2_ nucleation to occur. It is likely that HOIO_2_ formed in this way—or from the OIO + OH reaction—is taken up by iodine oxide clusters and aerosol.

Our laboratory results strongly support an atmospheric gas-to-particle conversion mechanism where the initial clustering steps are driven by I_*x*_O_*y*_ bimolecular and third-body reactions, both under dry and wet conditions. The mechanism of atmospheric IOP formation proposed in Fig. 7 is based on the set of reactions (Table 2) that best reproduces the time-resolved mass spectrometric data (Fig. 4) in the presence and absence of water, as described in the “Results” section. The IOP pyrolysis experiments show that HOI and HOIO_2_ are constituents of these particles, even though we did not observe them in the gas phase at room temperature, which implies that they are not indispensable IOP gas-phase precursors. This evidences the transformation of the I_*x*_O_*y*_ clusters into oxyacid-containing clusters within the flow tube residence time, which confirms earlier predictions about the composition of I_2_O_5_ IOPs in the MBL^28^. This scenario is represented in Fig. 7 by the hydration of the I_2_O_5_ units present in the iodine oxide clusters with the composition (I_2_O_5_)_*n*_OIO. We reported previously that the number and the size of IOPs reduces when they are formed in the presence of water^26^. Particle capillary collapse following water uptake could not alone explain those observations. The attachment of water molecules to molecular clusters may have the effect of reducing cluster formation and particle coagulation rates. At the molecular cluster level of the mass spectrometric observations discussed here, the addition of water results in a consistent reduction of the intensity of all masses without concomitant formation of products, which we have interpreted as inhibition of photofragmentation of larger clusters. We found previously^26,32^ that the key iodine oxide I_2_O_4_ forms a relatively stable complex with water, such that under the conditions prevailing in the MBL, a large fraction of I_2_O_4_ molecules would be hydrated as I_2_O_4_…H_2_O (> 40%). This could block the I_2_O_4_ halogen-bonding sites, and the subsequent necessary geometrical rearrangement for the reaction of I_2_O_4_ with itself or with I_x_O_y_ to proceed may result in barriers in the corresponding PES. The same may occur for larger molecular clusters. It should be noted that I_*x*_O_*y*_…H_2_O clusters are not observed in our experiments, which may be because they promptly dissociate following absorption of vacuum ultraviolet (VUV) photons.

A possible role of iodine in the formation and growth of new particles near the tropical tropopause has been proposed^9^. This would be favored by low water concentrations, but it must be noted that if the mixing ratios of I_*x*_O_*y*_ are low, the clustering process slows down compared to photolysis (photolysis lifetimes of tens of seconds^10,40^ versus reaction lifetimes of hours, Supplementary Table 5). In mid-latitude coastal regions, particle production probability shows a strong negative correlation with water vapor fluxes and relative humidity^31^. This is consistent with our laboratory observations of water slowing down IOP formation^26^, and not consistent with a mechanism which results in a positive dependence on water vapor. In the polar MBL, active iodine (IO_*x*_) mixing ratios are in general lower than in macroalgae-rich mid-latitude locations^5^, and consequently lower I_*x*_O_*y*_ mixing ratios should result in slower IOP formation rates (Supplementary Table 5). However, nucleation is likely to be favored not only by enhanced stability of molecular clusters at low temperature^26^ but also because the water content of the atmosphere is lower. In fact, nucleation events observed in the Arctic^21^ are coincident with periods of low RH.

Although we cannot rule out the formation of gas-phase HOIO_2_ (and HOIO) from I_2_O_*y*_ in the atmosphere (see lifetimes in Supplementary Table 5), we have shown that oxyacids are unlikely to be major nucleating species of IOPs. Moreover, we have evidence indicating that CI-API-ToF-MS IO_3_^−^ signals attributed to gas-phase HOIO_2_ prior to reaction using two reagent ions—nitrate and acetate—may require some re-interpretation (see Supplementary Note 2 and Supplementary Table 3). Besides collisional fragmentation issues of this technique^41^, IO_3_^−^ ions are exothermic reaction products of the dissociative charge transfer between I_2_O_*y*_ (*y* = 2, 3, 4) and NO_3_^−^ (and CH_3_COO^−^), and therefore it is likely that I_2_O_*y*_ contributes significantly to the IO_3_^−^ signal. The apparent paradox is that the IO_3_^−^ CI-API-ToF measurements^23^ remains a very useful proxy for tracking iodine nucleation in the field, because it may result from ionization of I_2_O_*y*_ and/or HOIO_2_ generated by I_2_O_*y*_ + H_2_O. Nevertheless, connecting the dots between these observations and those of precursors and smaller gas-phase species (I_2_, I, IO, HOI, OIO)^5^ is crucial to facilitating the development of a chemical mechanism that can be adapted for atmospheric modeling purposes. To our best knowledge, ours is the first complete iodine gas-to-particle conversion mechanism (Fig. 7) supported by direct experimental evidence. Future modeling work should adapt this scheme to carry out a global assessment of the contribution of iodine new particle formation to the atmospheric aerosol burden and its associated radiative forcing.

## Methods

### Pulsed laser and continuous broad band photolysis

A PLP system (Supplementary Fig. 1) was employed to generate iodine oxides from the 193-nm or 248-nm photolysis of I_2_/O_3_ mixtures in a 3.75-cm diameter tubular reaction cell. Alternatively, a Xenon lamp was used for continuous BBP of I_2_ in the presence of O_3_. A flow of 0.2–20 slm of N_2_ carrier gas (99.999%) was introduced in the reactor, depending on the target pressure. I_2_ molecules were entrained in the carrier flow by passing a smaller flow of carrier gas (10–500 sccm) through a heated, temperature-controlled flask containing I_2_ crystals. O_3_ was added by flowing O_2_ (10–200 sccm) through an electrical discharge. The excimer laser and the lamp beams were passed unfocussed through a quartz viewport along the tube main axis. An atmospheric pressure bubbler was employed to entrain water vapor into the carrier flow at approximately the equilibrium vapor pressure, resulting in a water mixing ratio of ~2% in the flow tube ([H_2_O] = 2.3 × 10^17^ molecule cm^−3^ at 350 torr and 295 K). The pressure in the reactor was set by a throttle valve placed between the reactor and an Edwards 80 roots blower—oil rotary pump combination. The flows were set using MKS calibrated mass flow controllers, and pressure was measured using a set of 10 Torr and 1000 Torr MKS Baratron pressure transducers. O_3_ and I_2_ concentrations were measured using a Herriott-type absorption cell situated upstream of the reactor^42^.

### Photoionization time-of-flight mass spectrometry

PI-ToF-Ms systems at Leeds University have been employed extensively to study reaction kinetics relevant to combustion-related and tropospheric organic and halogen chemistry^43–45^, and upper atmospheric chemistry of meteoric metals^46^. The first direct observation of I_*x*_O_*y*_ (*x* ≥ 2) gas-phase species was achieved using the older of these two systems^27^. The newer machine employed in this work has been described in detail in a previous publication^46^.

The gas in the reactor tube (Supplementary Fig. 1) is sampled on the axis via a skimmer cone with a 200-μm pinhole using a scroll pump-backed turbo-molecular pump. PI of molecules and clusters takes place in the ionization chamber (*P* = 10^−4^−10^−5^ Torr) before mass spectrometric detection. Alternatively, a differentially pumped roughing chamber can be inserted between the reactor and the PI chamber for experiments requiring high pressure (*P* > 20 Torr). In this configuration, the gas is sampled via a 500-μm pinhole into the roughing chamber, forming a high-density jet that is directed toward the 200-μm pinhole skimmer cone. The PI chamber is fitted with viewports, allowing a pulsed laser beam to be directed through the high-density region of the sampled gas jet. VUV ionization is achieved by tightly focusing in a rare-gas cell (Xe) the third harmonic of a Nd:YAG laser (Continuum Surelite 10-II), which produces VUV radiation by frequency tripling (118.2 nm, or equivalently 10.5 eV)^47,48^. The 118.2-nm radiation is delivered at the PI chamber by a waveguide, which is a ¼” glass tube with either constant inner diameter or tapered for enhanced PI efficiency. This technique shows an excellent detection limit for organic molecules such as acetone^43^ (10^11^ molecule cm^−3^ in a single accumulation in analog mode), which is used as a standard for optimizing the performance of the instrument. The conversion efficiency of frequency tripling in a rare gas is low (~10^−5^), and therefore VUV PI is a viable method for molecules with substantial PI cross-sections (>10^−18^ cm^2^ molecule^−1^). Possible interference from the 355-nm fundamental, if not separated from the VUV third harmonic, needs to be considered. This is important for molecules with UV–vis absorption bands, low ionization energy and high two-photon PI cross-sections. For example, water is detected at *m/z* = 18 (Fig. 1) using the 118.2-nm (10.5 eV) PI beam, even though its ionization potential is 12.621 eV. This most likely results from REMPI, where the intermediate state at 118.2 nm is the C electronic state or a Rydberg state of water^49^, and a 355-nm photon is enough for reaching the ground state of the ion. Although the PI beam is diverging in the ionization volume, this appears to be compensated by the high water concentrations employed. A 106.7 nm (11.6 eV) PI beam can also be generated by frequency tripling the second harmonic of a 532-nm pumped dye laser running on DCM dye. The power of this beam is approximately an order of magnitude lower than that of the 10.5 eV beam.

The positive ions resulting from PI of molecules in the sampled gas jet are accelerated toward the ToF-MS (Kore Technology) by a set of ion optics. This machine has been described in detail elsewhere^46^. A pre-amplifier coupled to the positive ion detector provides simultaneous analog and digital outputs. The analog output, which can be modulated linearly by varying the detector gain, is intended for registering large signals from species with high PI efficiency and/or high concentration. This output is recorded by using a digital oscilloscope (Picoscope 6000). The mass peaks appear in the time axis of the oscilloscope delayed with respect to the PI laser pulse by a characteristic time of flight. The peaks are then gate-integrated and passed on to a PC for further analysis. A limitation of this method is that strong signals cause detector overload after the corresponding peak during a significant lapse, which appears as a negative signal with respect to the spectrum baseline. Thus, peaks of other species arriving at the detector closely after the species causing a large peak are affected by this interference. Overloads can be eliminated by tuning down the detector gain, but this also reduces the sensitivity to small signals. To prevent detector overload by I^+^ and I_2_^+^, their mass peaks were gated out by pulsed biasing of the ion optics using a house-built dual-gating box.

The digital output is provided to a counting system (time-to-digital convertor, TDC) by means of a fast comparator. The Kore Technology TDC counts ions within a sequence of flight-time bins (0.5 ns) referred to the PI laser pulse. In this way, a histogram of counts versus time of flight, (i.e., a mass spectrum) is built. The counting method is suitable for capturing small signals (low concentration or low PI efficiency). The signal remains proportional to concentration for low counting rates. Although spectra measured with this technique are not affected by detector overload, for high counting rates, departure from linearity occurs and the peaks appear saturated.

Lasers and detectors are synchronized using a delay generator (Quantum Composers, 9518). In every measuring cycle, the delay generator triggers the Nd:YAG laser and after a few microseconds triggers the TDC or the scope to establish the time zero event of each measuring cycle. In addition, the delay generator triggers the excimer laser that initiates chemistry by photolyzing a radical precursor. The delay between the photolysis and the PI laser is scanned in order to sample reaction kinetics. In a typical digital experiment, 1000 digital spectra were accumulated for every reactant concentration and laser pulse delay, which at a 5-Hz laser-repetition rate takes 200 s. The built-in software of the MS-ToF does not handle synchronization of the delay generator and the TDC, and therefore the delays were set manually, at the cost of a poorer time resolution. Once the accumulation was complete, the delay between laser pulses and/or concentration of reagents was changed, and a new accumulation started. Kinetic experiments using the digital output are shown in Fig. 4 and Supplementary Fig. 3 (Exp. #1). Alternatively, the mass spectra in the analog mode were recorded by scanning automatically the delay between the lasers using a LabView program, which was coded to operate synchronously the delay generators and the digital oscilloscope. Typically, around 30 delay scans were average in analog mode. Kinetic experiments using the analog output are illustrated in Supplementary Fig. 3 (Exps. #2 and #3).

### Resonance fluorescence

Additional experiments to study the influence of water on the removal of atomic iodine were carried out with the Resonance and off-Resonance Fluorescence by Lamp Excitation (ROFLEX) machine^35^ in an atmospheric pressure BBP flow tube set up. A 3–15 slm flow of synthetic air carrying molecular iodine (~10^10^ molecule cm^−3^) and ozone (~10^12^ molecule cm^−3^) was passed to a 4-cm-diameter, 50-cm-long quartz tube where it was irradiated by a Xe arc lamp. The main flow and flow ratios were varied to keep the concentration of I_2_ and O_3_ constant while varying the residence time of the gas mixture on the tube. The gas mixture was sampled at the end of the flow tube by using a small aperture into the ROFLEX chamber at 40 Torr. Iodine atomic resonance fluorescence is excited by a temperature-stabilized iodine radiofrequency lamp, and collected perpendicularly by using a VUV-sensitive channel photomultiplier in counting mode. The data collected in these experiments is plotted in Supplementary Fig. 4. The analysis of this dataset using numerical kinetic modeling is explained below.

### Kinetic analysis

The processed PI-ToF-MS data consist of integrated peak signals (proportional to concentration) versus laser delay time in the case of PLP experiments, or a single mass spectrum in the case of BBP experiments, which correspond to a single time point, time being the residence time in the flow tube. The data obtained with the ROFLEX machine comprises the iodine atomic resonance fluorescence signal versus residence time in the flow tube.

Simple chemical systems can be described by sets of ordinary differential equations (ODEs) that are analytically solvable. In these cases, the observed data can be fitted to analytical expressions using a least-squares method, from which reaction rates can be extracted. This can be done for example in the case of the resonance fluorescence experiments if we assume that the only relevant reactions describing the behavior of atomic iodine are:

5 $${\mathrm{I}}_{\mathrm{2}} + {h\nu} \to {\mathrm{I}} + {\mathrm{I}}$$

6 $${\mathrm{I}} + {\mathrm{O}}_{\mathrm{3}} \to {\mathrm{IO}} + {\mathrm{O}}_{\mathrm{2}}$$

7 $${\mathrm{I}} \to {\mathrm{loss}}.$$

Because of the fast photolytic removal of I_2_, an effective iodine loss rate in the presence of water can be extracted by simply fitting exponential decays to the time traces and calculating the differences between the removal rates with and without water. This, however, would not allow the concentration of O_3_ to be estimated because IO reactions recycling atomic iodine are ignored:

8 $${\mathrm{IO}} + {\mathrm{IO}} \to {\mathrm{I}} + {\mathrm{OIO}}$$

9 $${\mathrm{IO}} + {{h\nu}} \to {\mathrm{I}} + {\mathrm{O}}$$

10 $${\mathrm{O}} + {\mathrm{O}}_{\mathrm{2}} + {\mathrm{M}} \to {\mathrm{O}}_{\mathrm{3}} + {\mathrm{M}}.$$

Multiplexed detection systems like the PI-ToF-MS provide simultaneous data from many molecules. To explain their kinetic traces, complex mechanisms with many reactions need to be constructed (e.g., Supplementary Tables 1 and 2). For these, the corresponding ODEs are coupled and not amenable to analytical solution. By combining an ODE numerical integrator and a nonlinear least-squares algorithm to fit simulated curves to the observed ones, some of the unknown reaction rates of the system may be determined, provided that the problem has enough degrees of freedom and that the free parameters are uncorrelated. When the initial conditions are known (concentration of precursors and laser energies), it is possible to derive the scaling factors of the signals observed by setting them as floating parameters. We use a standard integrator for stiff ODE problems (i.e., containing reaction rates spanning orders of magnitude) and a constrained nonlinear multivariable least-squares method from the Mathematics Matlab toolboxes^50^.

The left panel of Supplementary Fig. 4 shows the results obtained for the ROFLEX flow tube data with the numerical integration–nonlinear fitting analysis considering IO reactions. The right panel compares the effective removal rate constants obtained by analytical fitting and numerical integration–nonlinear fitting.

### Iodine oxide particle pyrolysis

The set up was modified to conduct a set of BBP-pyrolysis experiments by placing near the sampling point an iron wire shaped into a 10-mm diameter coil connected to a power supply by electrical feedthrough (Supplementary Fig. 2). The IOPs grew to a few nanometers^26^ in the flow tube at room temperature over ~3 s residence time. The carrier gas and particles passed through the first wire loop and were then sampled from the center of the coil in order to detect potential evaporation products. The color of the glowing filament suggested a temperature range of 1500–2500 K, although the gas temperature was presumably much lower. The gas spent a few tens of milliseconds in the hot region before sampling. Before adding water to the main flow, the current through the wire was changed until all I_*x*_O_*y*_ signals were removed by thermal dissociation, in order to minimize potential contributions of gas-phase reactions generating HOIO_2_ at high temperature.

### Electronic structure and master equation calculations

All gas-phase quantum-mechanical calculations reported in this work were performed using the Gaussian09^51^ software, and thermodynamic quantities are reported for the conditions of standard temperature (298.15 K) and pressure (1 atm).

The I_2_O_2_ + H_2_O ↔ HOIO + HOI and I_2_O_3_ + H_2_O ↔ HOIO_2_ + HOI reactions in the gas-phase (Supplementary Figs. 5 and 6, respectively) were explored using a high-level method employed in previous work for the I_2_O_4_ + H_2_O ↔ HOIO_2_ + HOIO (Supplementary Fig. 7) and I_2_O_5_ + H_2_O ↔ 2HOIO_2_ reactions^30^. The geometries of the stationary points on the PES of both reactions were first optimized at the M06-2X^52^ level of theory, using the aug-cc-pVDZ^53^ basis set for H and O, and the effective core potential LANL2DZ basis set for Iodine atoms (M06-2X/aug-cc-pVDZ+LANL2DZ). Normal-mode vibrational frequency analyses were carried out to ensure that the stable minima had all positive vibrational frequencies. Energies were refined at the CCSD(T)^54^ level of theory with the aug-cc-pVTZ+LANL2DZ basis set. For all reactions, unscaled vibrational frequencies calculated with the M06-2X/aug-cc-pVDZ+LANL2DZ method were used to estimate the zero-point energy (ZPE) corrections.

A detailed description of the methods employed in our Born–Oppenheimer Molecular Dynamics (BOMD) simulations can be found in a previous publication^30^. The droplet system contained 191 water molecules and one I_2_O_*y*_ (*y* = 2, 3, 4) molecule. The simulations were carried out in the constant volume and temperature (300 K) ensemble. The integration step was set as 1 femtosecond, which has been proven to achieve sufficient energy conservation in water systems^55–58^. The I_2_O_2_, I_2_O_3_, and I_2_O_4_ systems were simulated for 50, 80, and 33 picoseconds, respectively (Supplementary Videos 2, 3, and 4, respectively).

Estimates of vertical ionization and fragmentation energies of iodine oxides and oxyacids (Supplementary Table 2) as well as reaction enthalpies of relevant reactions (Table 1 and Supplementary Table 3) that are not available in the literature were obtained by using the B3LYP functional combined with the standard 6-311 + G(2d,p) triple-ζ basis set for O and H and an all-electron (AE) basis set for I^59^. This may be described as a supplemented (15s12p6d)/[10s9p4d] 6-311G basis, the [5211111111, 411111111, 3111] contraction scheme being supplemented by diffuse s and p functions, together with d and f polarization functions. The full basis set is given in Table XIII of Glukhovtsev et al.^59^ Following geometry optimizations of the reactant and product molecules and ions and the determination of their corresponding vibrational frequencies and (harmonic) ZPE, energies relative to the reactants were obtained. Geometry optimizations and energy calculations for most of the molecules listed in Table 1 are already available from our previous work^32,60^. The additional calculations in this work have been carried out for I_*x*_O_*y*_ with *y* = 3, 4 and HIO_z_ with *z* = 2, 3. Ionization energies and reaction enthalpies obtained at B3LYP/6-311 + G(2d, p) (+AE) level of theory compare reasonably well with experimental values and calculations at the higher level reported in the literature (see Table 1 and Supplementary Tables 2 and 3). This method has also been used to calculate the enthalpies of chemical ionization reactions of iodine oxides and oxyacids with nitrate, bromide and acetate ions generating IO^−^, IO_2_^−^, and IO_3_^−^ (Supplementary Table 3).

To determine rate constants from ab initio data, we have used the Master Equation Solver for Multi-Energy well Reactions (MESMER)^39,61^. The set of ro-vibrational energy levels in the ab initio PES are grouped into energy grains, whose populations are described by a system of coupled differential equations that account for collisional energy transfer and dissociation. The microcanonical rate coefficients of the unimolecular reactions that occur in each grain are calculated from the ab initio PES. For barrierless association reactions, the inverse Laplace transform method (ILT)^62,63^ is used to calculate the microcanonical association rates from an estimate of the high-pressure limiting-rate coefficient for the molecular association. The microcanonical dissociation rate coefficients are then determined by detailed balance. Collisional energy transfer probabilities are described by using the exponential down model. For further details, see Galvez et al.^32^ and references therein.

## Supplementary information

Supplementary material

Peer Review File

Description of Additional Supplementary Information

Supplementary Movie 1

Supplementary Movie 2

Supplementary Movie 3

Supplementary Movie 4

## Data Availability

The data that support the findings of this study are available from the corresponding authors on reasonable request.

## Code availability

The LabView and Matlab software tools written to respectively synchronize the experiments and analyze the kinetic data are available from the corresponding authors on reasonable request.
