# Supplementary material for: A gas-to-particle conversion mechanism helps to explain atmospheric particle formation through clustering of iodine oxides
Source: Nat Commun. 2020 Sep 9;11:4521. doi: 10.1038/s41467-020-18252-8 (PMC7481236; doi:10.1038/s41467-020-18252-8)
Supplement: Supplementary file 1 — Supplementary material [file 41467_2020_18252_MOESM1_ESM.pdf]

Supplementary material

A gas-to-particle conversion mechanism helps to explain atmospheric particle formation through clustering of iodine oxides

Juan Carlos Gomez Martin[1*], Thomas R. Lewis[2,3], Mark A. Blitz[3], John M.C. Plane[3], Manoj Kumar[4], Joe S. Francisco[4] and Alfonso Saiz-Lopez[2*]

[1] *Instituto de Astrofísica de Andalucía, CSIC, 18008 Granada, Spain.*

[2] *Department of Atmospheric Chemistry and Climate, Institute of Physical Chemistry Rocasolano, 28006, CSIC, Madrid, Spain.*

[3] *School of Chemistry, University of Leeds, LS2 9JT Leeds, UK.*

[4] *Department of Earth and Environmental Science and Department of Chemistry, University of Pennsylvania, Philadelphia, PA 19104-6323, USA*

**Correspondence to: J. C. Gómez Martín (jcgomez@iaa.es) and A. Saiz-Lopez (a.saiz@csic.es).*

Supplementary Note 1. ***Competition between iodine oxide and oxyacid cluster formation***

To explore the relative contribution of iodine oxides and oxyacids to the nucleation of IOPs, we have modelled a BBP flow tube experiments with longer residence time. The results shown in **Supplementary Figure 8** are for a flow tube residence time of 200 s. We have carried out two sets of simulations: one assuming that the source of $HOIO_2$ is the composite reaction I + H_2O + O_3 and another assuming that the source of both HOIO and $HOIO_2$ is the I_2O_4 + H_2O reaction, with the rate constants set at the upper limits determined experimentally (**Table 3**). The mechanism listed in **Supplementary Table 1** and **Table 1** is extended with HOIO and $HOIO_2$ aggregation reactions according to the scheme

$HOIO_x + HOIO_y \rightarrow H_2I_2O_{2+x+y}$ (x, y = 0, 1) (1)

$HOIO_x + H_2I_2O_y \rightarrow H_3I_3O_{3+x+y+z}$ (x = 0, 1; y = 5,6) (2)

$H_2I_2O_x + H_2I_2O_y \rightarrow H_4I_4O_{x+y}$ (x, y = 5, 6) (3)

No $HOIO_2$ addition and dehydration steps are considered because of the barrier in the $HOIO_2$ + $HOIO_2$ PES[1,2]. Calculated rate constants for the initial aggregation steps are listed in **Supplementary Table 4**. The HOIO self-reaction PES has a barrierless, exothermic pathway leading to the bimolecular product channel I_2O_3 + H_2O (**Table 1**). It is therefore a possible path from oxyacids back to oxides and an alternative explanation of a growing CI-API-ToF-MS IO_3^- signal with increasing H_2O concentration[3], since there is a likely contribution of I_2O_3 to this signal (see **Supplementary Note 2** and **Supplementary Table 3**). It can be seen that the formation of the HOIO…$HOIO_2$ aggregate is more favorable than the formation of the $HOIO_2$ dimer, while the following aggregation steps are assumed to proceed at the gas kinetics collisional rate constant. HOI is allowed to attach to hydroxide molecular clusters at 2×10^{-10} cm^{-3} s^{-1} $molecule^{-1}$. The oxide-oxyacid aggregation reactions forming I_2O_4…HIO_x (x = 1, 2) aggregates are also included, but these aggregates are not very stable. No other cross-over or hydrated clusters are considered.

The mixing ratio curves as a function of water concentration in **Supplementary Figure 8** correspond to major gas phase species. The total number density of molecular clusters with up to 9 iodine atoms descending from iodine oxides and oxyacids are displayed in the top panels to enable a comparison of the relative importance of oxides and oxyacids in the formation of clusters. The bottom panels show the iodine mass balance, i.e. the amount of

the initial atomic iodine in I_2 molecules which ends up as I_xO_y or HIO_x clusters. Despite the many assumptions involved in this exercise, a conclusion that can be drawn is that molecular clusters descending directly from HOIO and $HOIO_2$ can only become dominant at high water concentration (> 5×10^{16} molecule cm^{-3}) and if the direct precursor of these oxyacids is atomic iodine. If the precursor of oxyacids is an oxide, nucleation is started by iodine oxides. Simulations at lower I_2 photolysis rates yield similar I_xO_y/HIO_x ratios, because the $HOIO_2$ concentration depends on how much I_2O_4 is formed. Interestingly, even if $HOIO_2$ is not the species that drives nucleation, it is still a proxy for I_2O_4 and the evolution of its concentration would track that of particle formation events.

Supplementary Note 2. ***Interpretation of previous laboratory results and field data***

Observation of $HOIO_2$ by CI-API-ToF-MS was reported in laboratory experiments where a flow of synthetic air carrying I_2, O_3 and trace amounts of H_2O (i.e. no added water, RH < 1% or equivalently $x(H_2O)$ <0.03%, $[H_2O]$ < 7.7×10^{15} molecule cm^{-3}) was exposed to visible light[3]. No UV light was used, and therefore intervention of HO_x in the formation of $HOIO_2$ (e.g. via OIO + OH[4]) can be discounted. The IO^-, IO_2^- and IO_3^- signals with and without added water have been attributed to the chemical ionization by nitrate and acetate ions of the corresponding oxyacid neutral molecules HOI, HOIO and $HOIO_2$[3]. We have shown that gas-phase reactions forming oxyacids from atomic iodine and iodine oxides are very slow ($k \leq 5 \times 10^{-19}$ cm^3 s^{-1} $molecule^{-1}$). However, for reactor residence times of hundreds of seconds and sufficiently high water concentration, even these slow rate constants may result in $HOIO_2$ concentrations detectable by the sensitive nitrate CI technique (**Supplementary Figure 8**). We have hypothesized that wall reactions of iodine oxides with adsorbed H_2O may be an additional source of oxyacids in long residence time experiments and field instruments with long inlets. Thus, we have performed BOMD simulations to probe bond-forming and bond-breaking events over a finite time scale for I_2O_y on the air-water interface. **Supplementary Movies 2-4** show that I_2O_y (y = 2, 3, 4) do not react on time scales of tens of picoseconds, which indicates that HOIO and $HOIO_2$ would not be made by reactions of I_2O_y with H_2O adsorbed on the walls of the CI-API-ToF-MS inlet or the reactor inner surfaces. However, the possibility of higher iodine oxides reacting on the air-water interface[2] is intriguing and could be a source of both oxide and oxyacid clusters.

Besides thermochemical and kinetic obstacles for $HOIO_2$ formation, the interpretation of the

IO^-, IO_2^- and IO_3^- nitrate and acetate CI signals as oxyacids is problematic. **Supplementary Table 3** lists reaction enthalpies obtained from evaluated thermochemical data[5,6] complemented with our own ab initio calculations, showing that generation of IO^- and IO_2^- from reactions between HIO_x or I_xO_y and NO_3^-, Br^- and CH_3COO^- are generally endothermic. In contrast, dissociative charge transfer between $HOIO_2$ and NO_3^- is indeed exothermic. But IO_3^- can originate as well from dissociative charge transfer between I_2O_y (y=2, 3, 4) and NO_3^-, Br^- and CH_3COO^-. Although the IO_3^- CI signal has been shown to increase (sub-linearly) with water[3], which is an indication of $HOIO_2$ formation from a I_xO_y + H_2O slow reaction, it is very likely that I_2O_y contributes to it significantly. Many of the nitrogen-containing clusters observed in the CI-API-TOF-MS data can be identified as $I_xO_y.NO_3^-$ ($x \leq 3$, $y \leq 7$), which would confirm the presence of I_xO_y in this system.

The O/I ratio of 2.45 ± 0.05 of IOPs reported in our previous work under dry conditions[7] is consistent with clusters that start forming from molecules with O/I ≤ 2 and then grow at each step by I_2O_5 units (**Figure 1**). Due to this progressive increase of the I/O ratio, I_5O_{12} already shows an O/I ratio within the uncertainty range of IOPs. Under humid conditions, a more variable O/I ratio has been reported (2.2 - 2.6[3]), which indicates contribution of oxides and oxyacids to particle composition. Several studies report mass spectroscopic evidence about the composition of particles formed under different water vapor concentrations. The mass spectra of nanometer-sized IOPs generated in smog chamber studies by irradiation of CH_2I_2/Air/O_3 mixtures include the HIO_3^+ (electron impact[8]) and IO_3^- peaks (atmospheric pressure ionization, API[9]). These ion signals are consistent with the presence of $HOIO_2$ in IOPs. The use of the CH_2I_2 precursor provides a source of HO_x via UV photolysis of formaldehyde[10] generated by Crigee-iodine chemistry [11], which could result in the formation of $HOIO_2$ under dry conditions. The API IO_3^- signal is also compatible with iodine oxides[9] . These factors possibly prevented a stronger contrast at m/z = 176 between humid and dry runs. More importantly, the high temperature employed in these works to vaporize particles (823-873 K) was possibly too high to allow the survival of enough $HOIO_2$[8].

The recent observation by CI-API-ToF-MS of ion clusters compatible with oxyacid composition alongside signals attributed to $HOIO_2$, and the increase of these signals with water, have been interpreted as evidence of $HOIO_2$ homogeneous nucleation. The composition of the particles, therefore, is proposed to be the same as that of the gas phase molecules. The PI positive ion

mass spectra recorded in the presence of water under a variety of conditions (**Figure 2**) appear to show a different picture, where the larger clusters are not grown by sequential addition of the smaller ones, and where water only reduces the signal without creating new peaks. Comparison between mass spectra obtained by different sampling and ionization techniques is not straightforward, but a plausible explanation is the much longer residence time and lower I_xO_y concentration in the CI experiments and field measurements, which may create a more favorable situation for slow water reactions. Reactions between I_xO_y and H_2O may be even slower than the upper limits presented here, but the much longer residence time in the CI experiments and field measurements may allow for attachment of water and oxyacids to pre-existing clusters. Moreover, fragmentation mechanisms of a completely different nature are at play in both systems. Collision-induced fragmentation has been identified as a problem obscuring the interpretation of API-MS data[12]. In fact, the CI mass spectra show a large number of peaks which are unassigned or do not fit into the proposed $HOIO_2$ nucleation scheme. A striking similarity between the PI and CI spectra is that the groups of peaks with even numbers of iodine atoms are separated by I_2O_5 units (334 Da), and so are the groups with odd numbers of iodine atoms, which once again shows that I_2O_5 (or its hydrated form $HOIO_2$) is the backbone composition of IOPs.

Supplementary Figure 1

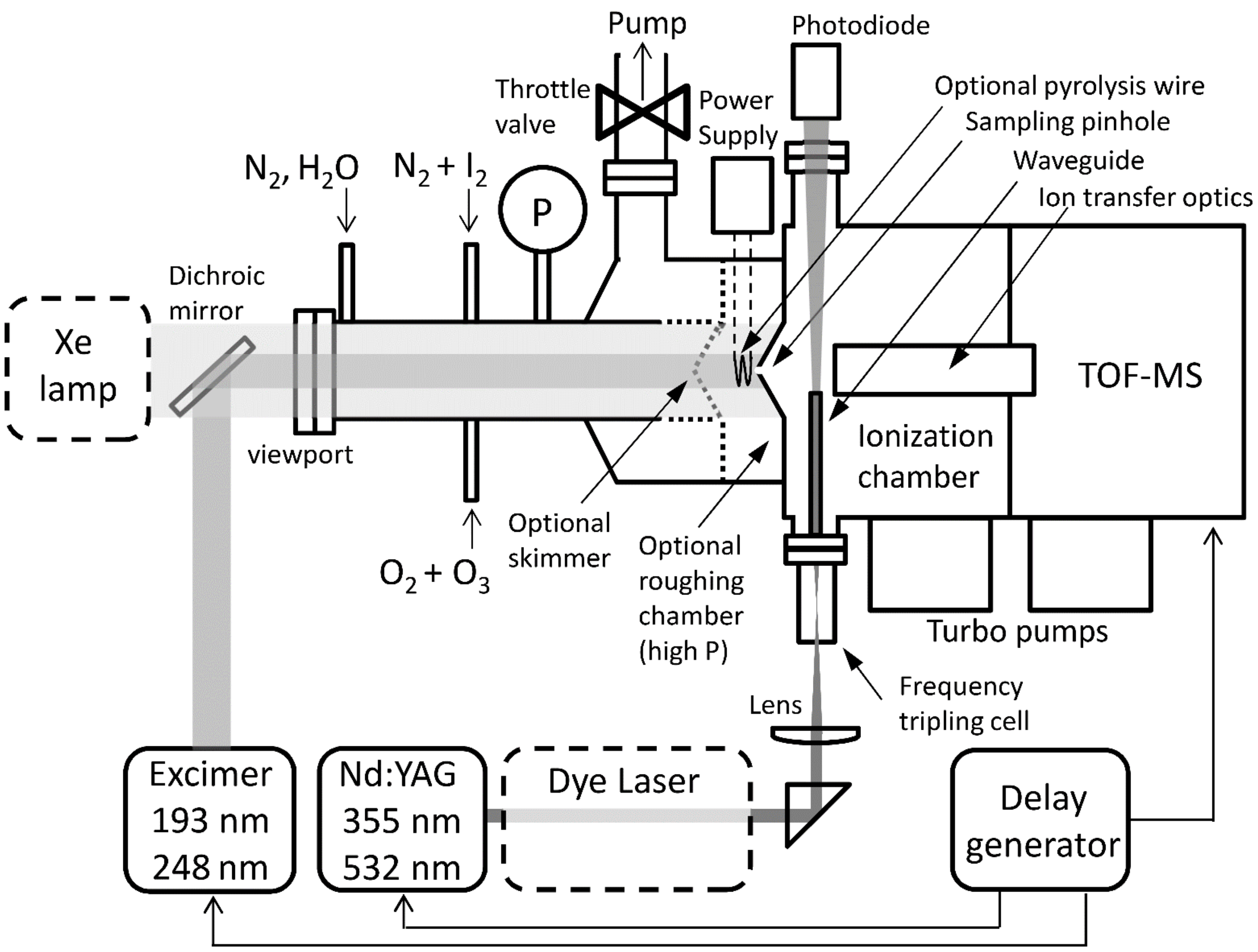

***Supplementary Figure 1*. Experimental set up employed in this work**. *Pulsed Laser Photolysis (PLP) experiments*: A dichroic mirror is used to steer the excimer beam (193 nm or 248 nm) into the flow tube. *Broad Band Photolysis (BBP) experiments*: the dycroic mirror is removed and a xenon lamp is used instead. High pressure experiments: the tube is shortened and a differentially pumped roughing chamber (dotted lines) is insterted between the experiment and the ionization chamber. *Pyrolysis experiments*: a hot filament (thin dashed line) is exposed to the flow near the sampling point (see **Supplementary Figure 2**). Two ionisation energies are available: (*a*) tripplig the third harmonic (354.7 nm) of the Nd:YAG laser in a rare gas (Xe), resulting in 118.2 nm photons (10.5 eV) and (*b*) using the second harmonic (532 nm) of the Nd:YAG laser to pump a dye laser running on a DCM special solution in ethanol, to obtain a 322.8 nm beam, which is then tripled to obtain 106.7 nm photons (11.6 eV).

Supplementary Figure 2

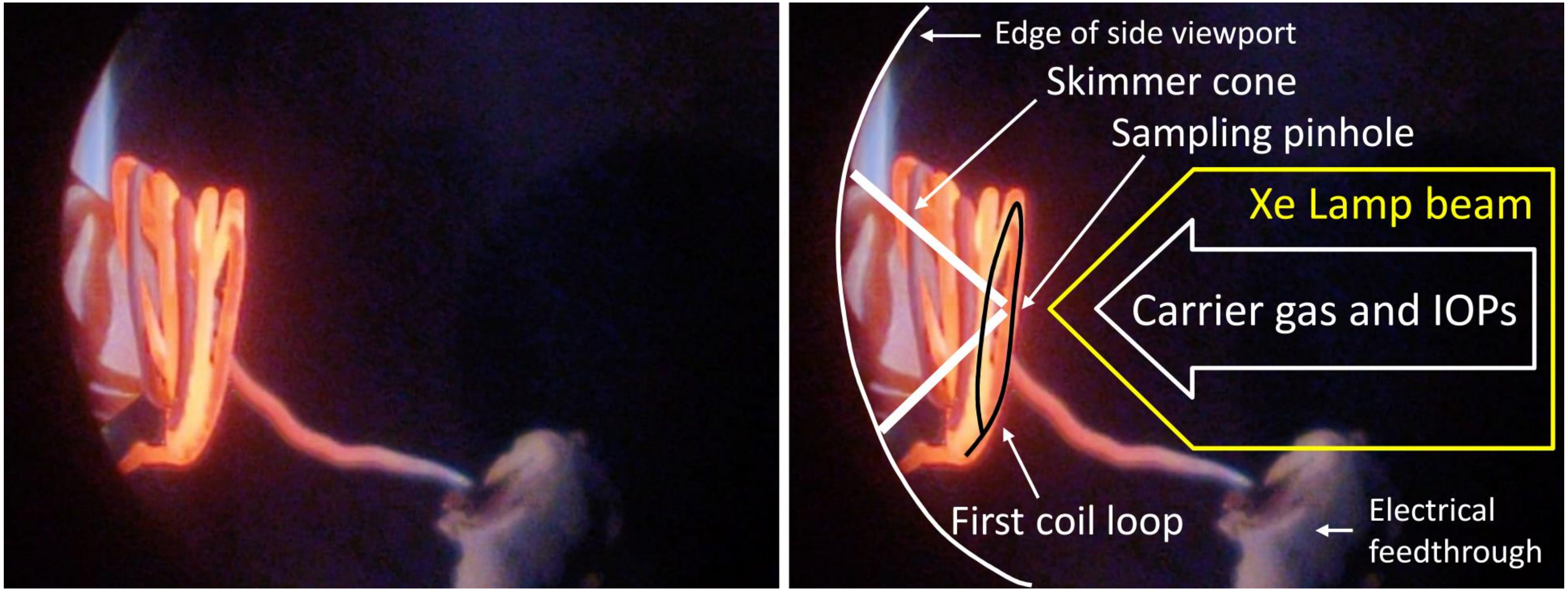

Supplementary Figure 2. **Pyrolysis set-up.** Left: view of the resistively heated coil placed around the sampling point in the pyrolysis experiments. The picture was taken from a viewport perpendicular to the flow tube, situated where the power supply of the coil is depicted in **Supplementary Figure 1**. Right: same picture with annotations indicating different elements of the set up and the sampling geometry.

Supplementary Figure 3

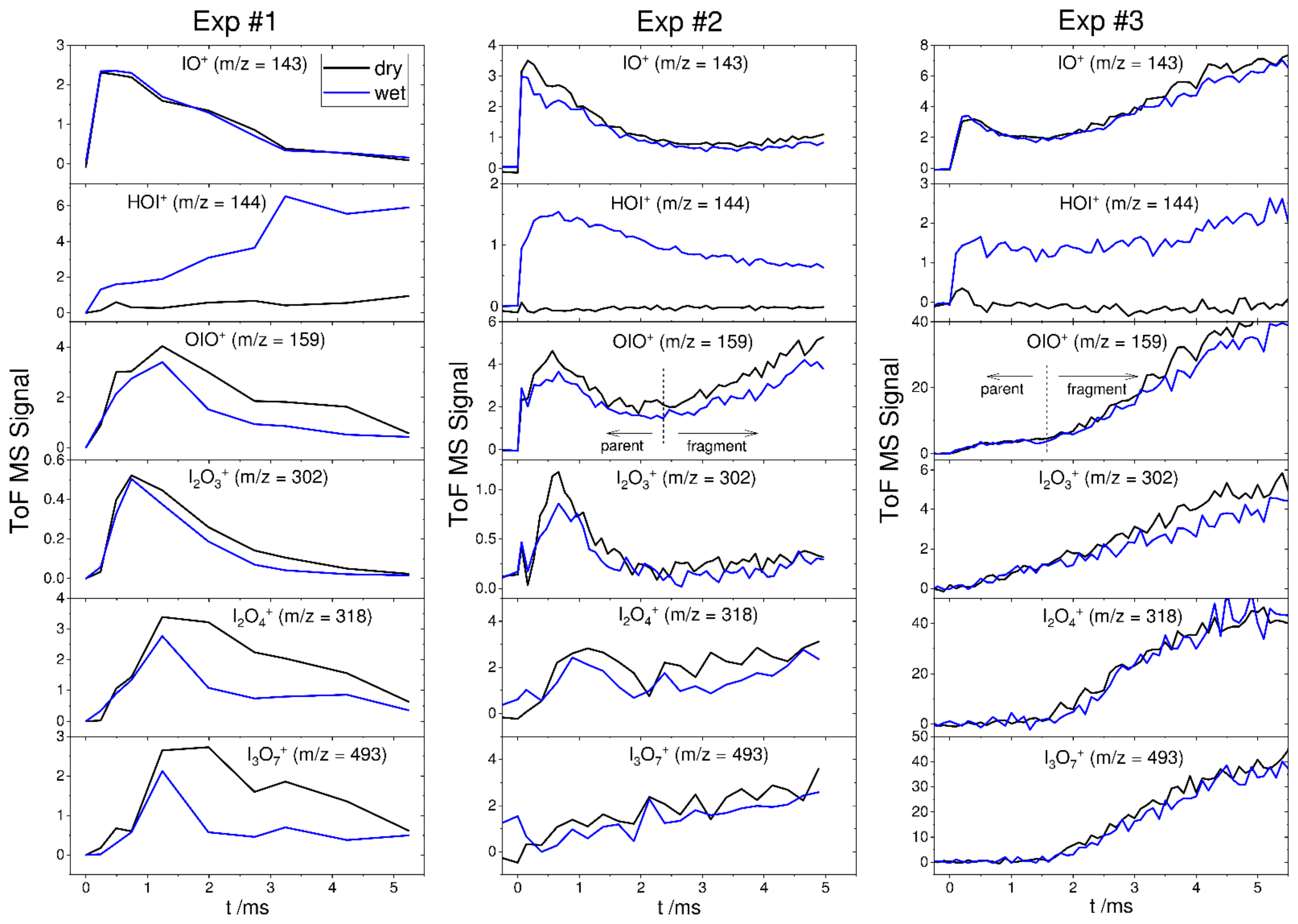

Supplementary Figure 3**. Kinetic traces**. Recorded from three different PLP experiments initiated by 248 nm photolysis of O_3 in the presence of I_2 at 10 Torr (PI 10.5 eV). The blue curves correspond to experiments where water was added actively to the flow (mixing ratio of 2%), while the black curves correspond to dry experiments. *Experiment #1*: digital model, manual PLP-PI delaying, weaker ionization. *Experiments #2 and #3*: analog mode, automatic PLP-PI delaying, higher I_xO_y concentrations. The three experiments have a different PI beam delivery geometry and energy, resulting in different fragmentation of higher oxides. In experiment #3, with the highest concentration of I_xO_y, the true signals are swamped by the fragmentation signal. The addition of water, besides the formation of HOI (second row), appears to reduce the extent of fragmentation rather than causing chemical removal, since new products are not observed.

Supplementary Figure 4

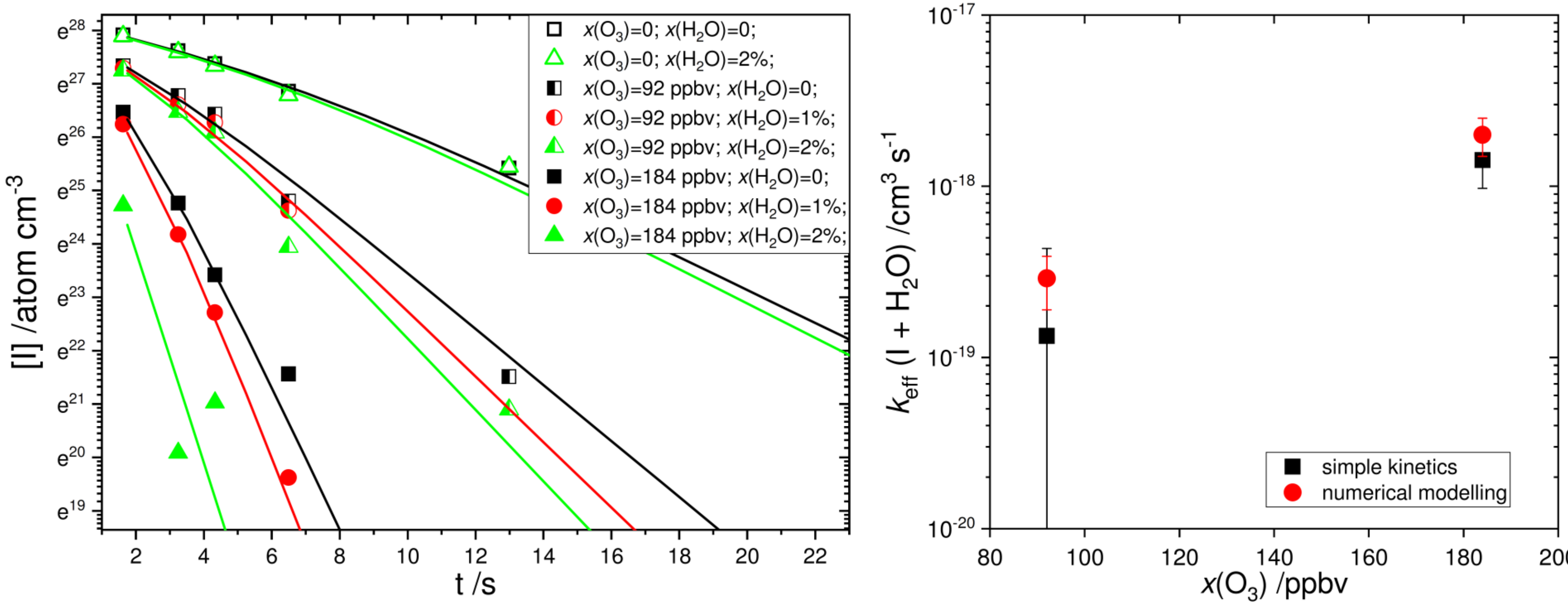

Supplementary Figure 4. **ROFLEX measurements**. Removal of atomic iodine in the presence of O_3 and water vapor as observed in a BBP flow tube experiment at 760 Torr by atomic resonance fluorescence using the ROFLEX instrument. Left panel: concentration versus residence time traces and numerical fits to the data. The residence time is varied by changing the main carrrier flow while keeping the ratio to the I_2 carrier flow constant. Water concentrations were changed by passing the full carrrier flow or just half of it through a water bubbler. Ozone concentrations (given as mixing ratios in parts per billion in volume, ppbv) were varied by changing the distance between a Hg pen-ray lamp and a flow of O_2 through a small photolysis cell with quartz windows. Two types of kinetic analysis were performed: (i) simple exponential fits to the decay traces allow calculating the effective removal rate by water, by taking the differences between the rates with and without ozone and water and (b) numerical modelling and fitting of the data, which in addition enables estimating the ozone concentration. The resulting effective rate constants are plotted in the right panel. There apppears to be a slight enhancement of the removal rate of iodine in the presence of water. However, the effective rate constant under typical ozone and water concentrations is very small, of the order of 3×10^{-19} cm^3 s^{-1} molecule^{-1}.

Supplementary Figure 5

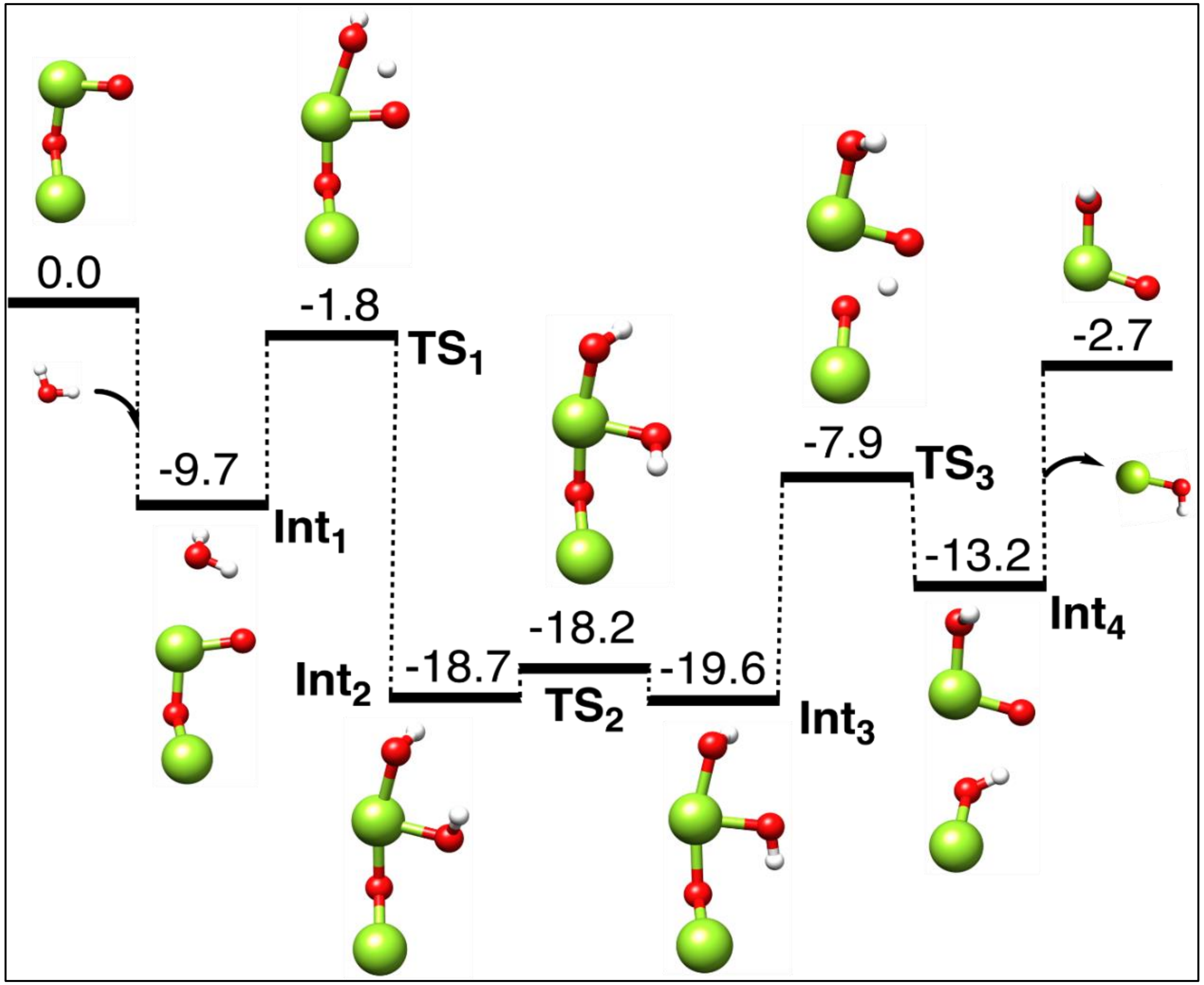

Supplementary Figure 5. **Potential energy surface of the I_2O_2 + H_2O reaction**. CCSD(T)/aug-cc-pVTZ+LANL2DZ//M06-2X/aug-cc-pVDZ+LANL2DZ calculated reaction profile for $I_2O_2 + H_2O \rightarrow$ HOIO + HOI. The zero-point-corrected electronic energies are relative to the separated constituents and are given in units of kcal mol^{-1}. The zero-point correction term was calculated at the M06-2X/aug-cc-pVDZ+LANL2DZ level of theory.

Supplementary Figure 6

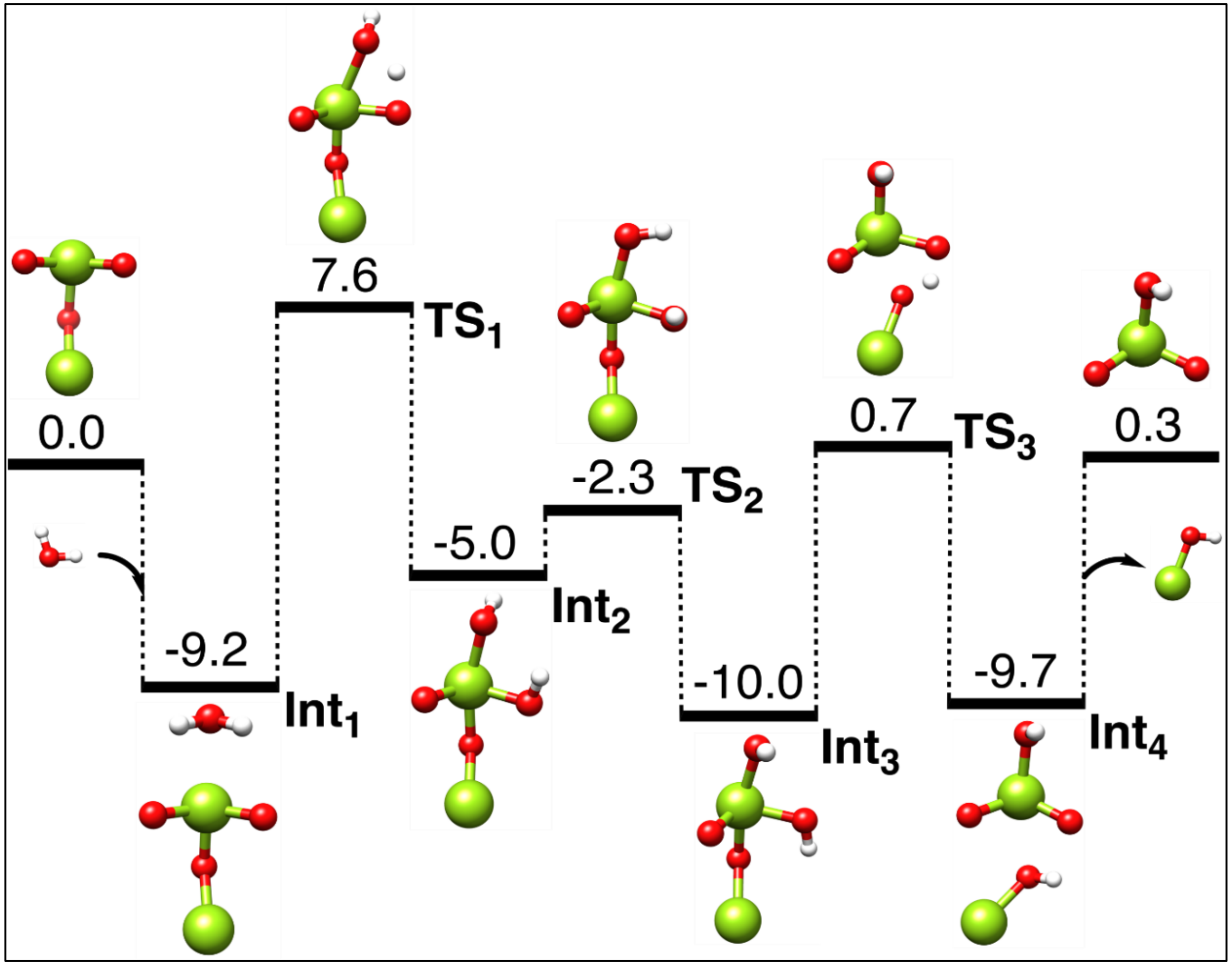

Supplementary Figure 6. **Potential energy surface of the I_2O_3 + H_2O reaction**. CCSD(T)/aug-cc-pVTZ+LANL2DZ//M06-2X/aug-cc-pVDZ+LANL2DZ calculated reaction profile for $I_2O_3 + H_2O \rightarrow HOIO_2$ + HOI. The zero-point-corrected electronic energies are relative to the separated constituents and are given in units of kcal mol^{-1}. The zero-point correction term was calculated at the M06-2X/aug-cc-pVDZ+LANL2DZ level of theory.

Supplementary Figure 7

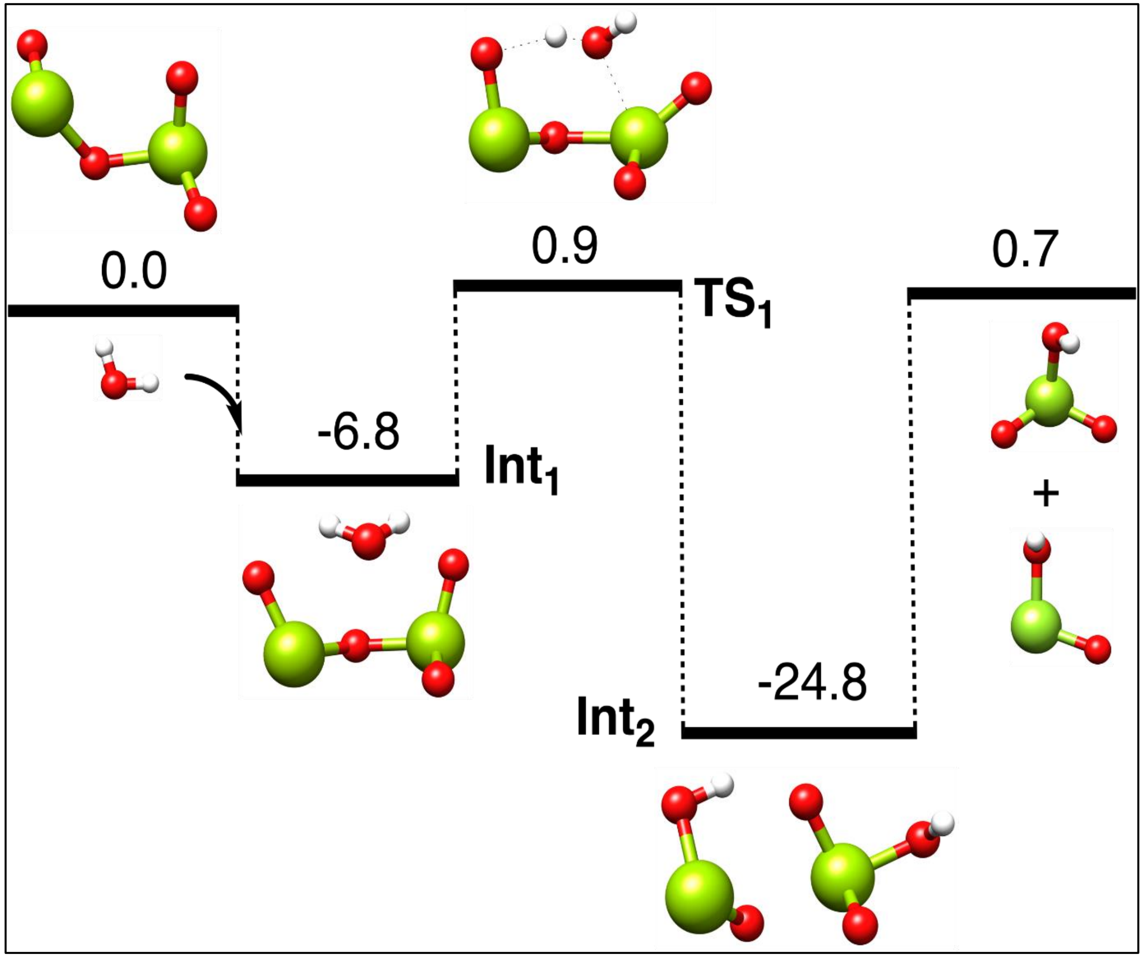

Supplementary Figure 7. **Potential energy surface of the I_2O_4 + H_2O reaction**. CCSD(T)/aug-cc-pVTZ+LANL2DZ//M06-2X/aug-cc-pVDZ+LANL2DZ calculated reaction profile for the *concerted* I_2O_4 + H_2O reaction[2], which results in the formation of $HOIO_2$ and HOIO. The zero-point-corrected electronic energies are relative to the separated constituents and are given in units of kcal mol^{-1}. The zero-point correction term was calculated at the M06-2X/aug-cc-pVDZ+LANL2DZ level of theory.

Supplementary Figure 8

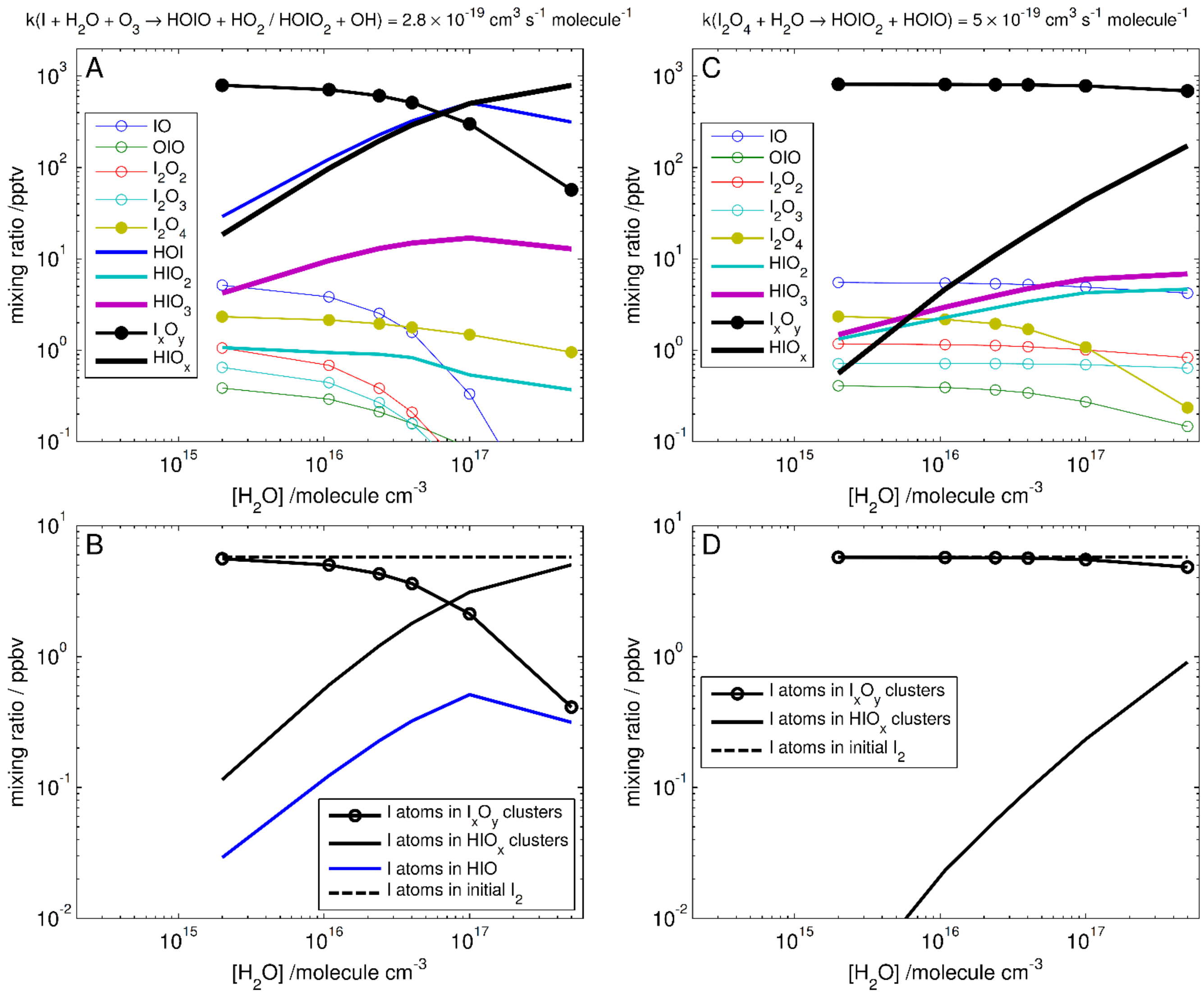

***Supplementary Figure 8*. Simulated mixing ratios as a function of water concentration for a flow tube BBP experiment**. Conditions: visible I_2 photolysis, P = 760 Torr, residence time = 200 s, $[I_2]_0 = 7.2 \times 10^{10}$ molecule cm^{-3}, $[O_3]_0 = 1.5 \times 10^{12}$ molecule cm^{-3}). Symbols refer to oxides and solid lines to oxyacids. Black lines and symbols refer to the sum of clusters with more than 3 iodine atoms. The bottom panels show the iodine atom budget. The source of oxyacids is assumed to be the composite reaction $I + H_2O + O_3 \rightarrow HOIO + HO_2$ / $HOIO_2 + OH$ (panels *a* and *b*) or the reaction I_2O_4 + H2O $\rightarrow HOIO_2 + HOIO$ (panels *c* and *d*) . The rate constants of these reactions are set at the upper limits determined experimentally The reacions compiled in **Supplementary Table 1** and **Table 2** are considered for iodine oxides. For oxyacids, a scheme of stepwise addtion of HOIO and $HOIO_2$ is assumed (k = 2×10^{-10} molecule cm^{-3} for all reactions). No $HOIO_2$ addition and dehydration steps[3] are considered because of the barrier in the $HOIO_2 + HOIO_2$ PES[1]. The production of HO_x in the $I+H_2O+O_3$ results in high HOI concentration (green line in panel *a*).

Supplementary Tables

Supplementary Table 1. IOP-source chemical mechanism.

**Reaction**	***k* (295 K, 10 Torr) /cm³ molecule⁻¹ s⁻¹**	***k* (295 K, 350 Torr) /cm³ molecule⁻¹ s⁻¹**	**Ref.**[a]
$I_2 + h\nu_{193, VIS} \rightarrow I + I$ [b]			
$O_3 + h\nu_{248} \rightarrow O(^1D) + O_2(a^1\Delta)$			
$O(^1D) + N_2 \rightarrow O(^3P) + N_2$	3.1×10^{-10}		5
$O(^3P) + I_2 \rightarrow IO + I$	1.25×10^{-10}		13
$O(^3P) + IO \rightarrow I + O_2$	1.4×10^{-10}		13
$I + O_3 \rightarrow IO + O_2$	1.26×10^{-12}		13
$IO + IO \rightarrow OIO + I$	7.6×10^{-11}	4.3×10^{-11}	13
$IO + IO \rightarrow IOIO$	2.4×10^{-11}	5.7×10^{-11}	13
$IO + OIO \rightarrow I_2O_3$	2×10^{-11}	1.3×10^{-10}	13
$OIO + OIO \rightarrow I_2O_4$	1×10^{-11}	6.9×10^{-11}	13
$O(^1D) + H_2O \rightarrow OH + OH$	2×10^{-10}		5
$OH + I_2 \rightarrow HOI + I$	2.1×10^{-10}		13
$IO + h\nu_{VIS} \rightarrow I + O$			
$OIO + h\nu_{VIS} \rightarrow I + O_2$			

[a] Evaluated rate constants for atmospheric iodine chemistry modelling (see Saiz-Lopez et al. [13] and references therein). Reactions involving other species from the JPL-NASA evaluation[5].

[b] PLP experiments use an excimer laser at 193 (I_2 photolysis at the Cordes band system[14]) or 248 nm (O_3 photolysis at the Hartley band). BBP experiments use a visible broad band source that mainly photolyzes I_2 in its visible absorption spectrum around 500 nm (σ_{I2}(500nm)=2.18×10^{-18} cm^2 molecule^{-1} [15]), while the photolysis of O_3 at the Chappuis band is comparatively small (maximum: σ_{O3}(600 nm)=5.2×10^{-21} cm^2 molecule^{-1} [16]).

Supplementary Table 2. Calculated photoionization and photofragmentation energies of iodine oxides and oxyacids

Photo-ionization	IP / eV cal.	IP /eV cal. literature [17]	IP /eV exp.	Ref.
$IO \rightarrow IO^+ + e^-$	9.9	9.6	9.745 ± 0.017	[18]
$OIO \rightarrow OIO^+ + e^-$	10.3	9.7	9.80 ± 0.10	[19]
$IOI \rightarrow IOI^+ + e^-$	9.9	10.4	<9.3	[19]
$IIO \rightarrow IIO^+ + e^-$	9.0	9.0		
$IOIO \rightarrow IOIO^+ + e^-$	9.1	9.1		
$I_2O_3 \rightarrow I_2O_3^+ + e^-$	10.2	10.0	9.90 ± 0.03	[19]
$I_2O_4 \rightarrow I_2O_4^+ + e^-$	9.4	9.3		
$I_2O_5 \rightarrow I_2O_5^+ + e^-$	10.8	11.4		
$I_3O_6 \rightarrow I_3O_6^+ + e^-$	10.0	9.1		
$I_3O_7 \rightarrow I_3O_7^+ + e^-$	10.6	8.7		
$HOI \rightarrow HOI^+ + e^-$	9.9		9.81 ± 0.02	[20]
$HOIO \rightarrow HOIO^+ + e^-$	9.3			
$(HO)_2IO \rightarrow (HO)_2IO^+ + e^-$	10.1		[b]	
$HOIO_2 \rightarrow HOIO_2^+ + e^-$	11.1	11.3 [a]	[c]	
$HI_2O_5 \rightarrow HI_2O_5^+ + e^-$	10.6		[b]	
**Fragmentation**	**Δ*H*r /eV cal.**	**Fragmentation**	**Δ*H*r /eV cal**	
$IOIO \rightarrow IOI^+ + O + e^-$	11.9	$I_3O_7 \rightarrow I_3O_6^+ + O + e^-$	12.4	
$IOIO \rightarrow IO^+ + IO + e^-$	10.7	$I_3O_7 \rightarrow I_2O_4^+ + IO_3 + e^-$	10.8	
$IOIO \rightarrow OIO^+ + I + e^-$	11.1	$I_3O_7 \rightarrow OIO^+ + I_2O_5 + e^-$	10.7	
$IOIO \rightarrow I^+ + OIO + e^-$	11.5	$I_4O_6 \rightarrow I_3O_6^+ + I + e^-$	11.6	
$I_2O_3 \rightarrow OIO^+ + IO + e^-$	11.6	$I_4O_6 \rightarrow I_2O_3^+ + I_2O_3 + e^-$	10.8	
$I_2O_3 \rightarrow IO^+ + OIO + e^-$	11.2	$I_4O_7 \rightarrow I_3O_7^+ + I + e^-$	11.2	
$I_2O_3 \rightarrow IOIO^+ + O + e^-$	12.2	$I_4O_7 \rightarrow I_3O_6^+ + IO + e^-$	11.9	
$I_2O_4 \rightarrow OIO^+ + OIO + e^-$	11.1	$I_4O_7 \rightarrow I_2O_3^+ + I_2O_4 + e^-$	10.9	
$I_2O_4 \rightarrow I_2O_3^+ + O + e^-$	12.1	$I_4O_7 \rightarrow I_2O_4^+ + I_2O_3 + e^-$	10.1	
$I_2O_5 \rightarrow OIO^+ + IO_3 + e^-$	12.1	$I_4O_8 \rightarrow I_3O_7^+ + IO + e^-$	11.0	
$I_3O_6 \rightarrow I_2O_3^+ + IO_3 + e^-$	11.0	$I_4O_8 \rightarrow I_2O_4^+ + I_2O_4 + e^-$	9.8	
$I_3O_6 \rightarrow I_2O_4^+ + OIO + e^-$	10.2	$HOIO \rightarrow IO^+ + OH$	11.4	
$I_3O_6 \rightarrow OIO^+ + I_2O_4 + e^-$	11.2	$HOIO_2 \rightarrow OIO^+ + OH$	12.45	
$I_3O_6 \rightarrow IO^+ + I_2O_5 + e^-$	10.3			

[a] The ionization energy of $HOIO_2$ was determined by Gomez Martin et al.[17] at the CCSD(T)//MP2/aug-cc-pVTZ (AREP) level of theory[21], but was not reported in Table 1 of that paper.

[b] Correspond to peaks seen in this work

[c] Has been detected by PI-ToF-AMS at 11 eV and higher ionization energies [19].

Supplementary Table 3. Reaction enthalpies for iodine oxides and oxyacids for three common CI sources (nitrate, bromide and acetate ions).

**Analyte ↓**	**Ion source →**	**NO_3^- [22]**			**Br^- [22,23]**			**CH_3COO^- [24]**		
	**Ion detection →**	**IO^-**	**IO_2^-**	**IO_3^-**	**IO^-**	**IO_2^-**	**IO_3^-**	**IO^-**	**IO_2^-**	**IO_3^-**
IO	evaluated	148 ± 3	86 ± 3	-31 ± 11	93 ± 2			82 ± 4		
OIO	evaluated		131 ± 4	-85 ± 11		75 ± 3			64 ± 4	72 ± 11
	ab initio[a]			*12*						*156*
I_2O	evaluated		118 ± 10	4 ± 14						
I_2O_2	evaluated		56 ± 15	-95 ± 19		-32 ± 15				
I_2O_3	evaluated			-32 ± 16		120 ± 11	-121 ± 14			
	ab initio[b]		109	-10		188	-101		84	-112
I_2O_4	evaluated						-109 ± 18			
	ab initio[b]			2.1			-76			-101
HOI	evaluated	132 ± 5			138 ± 4			35 ± 5		
	ab initio[c]	**124**								
HOIO	evaluated		65 ± 3			70 ± 2			-32 ± 3	
	ab initio[b,c]		65 **57**			67			-36	
$HOIO_2$	evaluated			-35 ± 10			-30 ± 10			-132 ± 11
	ab initio[c]			**-23**						

[a] wB97xd/6-311+G(2df,2p) Sipilä et al.[3] (Italics)

[b] b3lyp/6-311+G(2d,p) with All electron iodine basis set[25] This work (normal typescript).

[c] CCSD(T)/aug-cc-pV5Z//PBE0/aug-cc-pVQZ with ECP28MDF effective core for iodine. Sipilä et al.[3] (bold)

Supplementary Table 4. Calculated reaction enthalpies (i.e. bond energies), rate constants and dissociation rates of possible oxyacid and oxide-oxyacid-aggregation and dissociation reactions (T = 295 K, 760 torr)

Reaction	**ΔH_r (298 K)**	k
$HOIO + HOIO_2 \rightarrow HOIO...HOIO_2$	-104 kJ mol^{-1} [2]	6.7×10^{-11} cm^3 s^{-1}
$HOIO...HOIO_2 \rightarrow HOIO + HOIO_2$		0.8 s^{-1}
$HOIO_2 + HOIO_2 \rightarrow HOIO...HOIO_2$	-84 kJ mol^{-1} [1]	1.1×10^{-11} cm^3 s^{-1}
$HOIO...HOIO_2 \rightarrow HOIO + HOIO_2$		1388 s^{-1}
$I_2O_4 + HOIO \rightarrow I_2O_4...HOIO$	-64 kJ mol^{-1} [2]	3.7×10^{-12} cm^3 s^{-1}
$I_2O_4...HOIO \rightarrow I_2O_4 + HOIO$		1×10^{6} s^{-1}
$I_2O_4 + HOIO_2 \rightarrow I_2O_4...HOIO_2$	-94 kJ mol^{-1} [2]	1.0×10^{-10} cm^3 s^{-1}
$I_2O_4...HOIO_2 \rightarrow I_2O_4 + HOIO_2$		270 s^{-1}

Supplementary Table 5. Estimated atmospheric first order lifetimes of I_2O_4 by reaction with I_xO_y and with H_2O (lower limits), and of HIO_x by reaction with $HOIO_2$ [a].

			$I_2O_4 + I_xO_y$		$I_2O_4 + H_2O$			$HOIO_2 + HIO_x$	
	T /K	P /hPa	$x(I_xO_y)$ /pptv [b]	τ /min	RH (%)	*x* (H_2O) (%)	τ /min	*x* ($HOIO_2$) (%) [c]	τ /min
**Coastal MBL Mid-latitudes**	293	1013	10	0.3	100	2.3	> 0.1	10	0.3
			20	*0.2*					
	283	1013	10	0.3	80	1.0	> 0.1	10	0.3
			20	*0.2*					
**Polar MBL**	273	1013	0.5	6.2	100	0.6	> 0.2	0.1	31
			5	*0.6*				*1*	*3.1*
	253	1013	0.5	5.7	100	0.1	> 0.9	0.1	29
			5	*0.6*				*1*	*2.9*
**UT-LS**	273	500	0.02	314	8	0.1	> 2.5		
			0.2	*31*	8				
	233	250	0.02	536	13	0.01	> 43		
			0.3	*36*	13				
	213	125	0.02	981	7	0.001	> 784		
			0.4	*49*	7				

[a] The estimated effective rate constant for I_2O_4 based on its observed phenomenological loss in laboratory experiments at 350 Torr ($k' \sim 2000$ s^{-1}), assuming that it reacts with I_xO_y ($[I_xO_y] \sim 10^{13}$ cm^{-3}) according to **Table 2**.
The rate constant used for $HOIO_2 + HIO_x$ has been calculated with MESMER (**Supplementary Table 4**) .The rate constants for water reactions are set at the upper limits determined in this work: $k(I_2O_4 + H_2O) = 5 \times 10^{-19}$ cm^3 s^{-1} $molecule^{-1}$, and $k(I + H_2O + O_3) = 2.8 \times 10^{-19}$ cm^3 s^{-1} $molecule^{-1}$.
[b] Mixing ratios of I_xO_y estimated from atmospheric modelling of iodine chemistry in the polar and coastal MBL[26,27] and the tropical upper troposphere[13]. For the polar MBL and the Upper Troposphere-Lower Stratosphere, two scenarios $x(I_xO_y)$ scenarios are given for fast I_xO_y photolysis (numbers in normal typescript) and no I_xO_y photolysis (numbers in italics)[13].
[c] Reported peak mixing ratios of $HOIO_2$[3] in Antarctica (normal typescript) and Greenland (italics).

Supplementary References

1 Khanniche, S., Louis, F., Cantrel, L. & Černušák, I. Computational study of the I2O5+H2O=2 HOIO2 gas-phase reaction. *Chem. Phys. Lett.* **662**, 114-119, doi:http://dx.doi.org/10.1016/j.cplett.2016.09.023 (2016).

2 Kumar, M., Saiz-Lopez, A. & Francisco, J. S. Single-Molecule Catalysis Revealed: Elucidating the Mechanistic Framework for the Formation and Growth of Atmospheric Iodine Oxide Aerosols in Gas-Phase and Aqueous Surface Environments. *J. Am. Chem. Soc.* **140**, 14704-14716, doi:10.1021/jacs.8b07441 (2018).

3 Sipilä, M. *et al.* Molecular-scale evidence of aerosol particle formation via sequential addition of HIO3. *Nature* **537**, 532-534, doi:10.1038/nature19314 (2016).

4 Plane, J. M. C., Joseph, D. M., Allan, B. J., Ashworth, S. H. & Francisco, J. S. An Experimental and Theoretical Study of the Reactions OIO + NO and OIO + OH. *J. Phys. Chem. A* **110**, 93-100 (2006).

5 Burkholder, J. B. *et al.* Chemical Kinetics and Photochemical Data for Use in Atmospheric Studies, Evaluation No. 18 (JPL-NASA, Pasadena, 2015).

6 *NIST Chemistry WebBook, NIST Standard Reference Database Number 69*. (National Institute of Standards and Technology, 2019).

7 Saunders, R. W., Mahajan, A. S., Gómez Martín, J. C., Kumar, R. & Plane, J. M. C. Studies of the Formation and Growth of Aerosol from Molecular Iodine Precursor. *Z. Phys. Chem.* **224**, 1095-1117 (2010).

8 Jimenez, J. L. *et al.* New particle formation from photooxidation of diiodomethane (CH2I2). *J. Geophys. Res.* **108**, 4318 (2003).

9 Hoffmann, T., O'Dowd, C. D. & Seinfeld, J. H. Iodine oxide homogeneous nucleation: An explanation for coastal new particle production. *Geophys. Res. Lett.* **28**, 1949-1952 (2001).

10 Seinfeld, J. S. & Pandis, S. N. *Atmospheric Chemistry and Physics*. (Wiley-Interscience, 1998).

11 Stone, D., Blitz, M., Daubney, L., Ingham, T. & Seakins, P. CH2OO Criegee biradical yields following photolysis of CH2I2 in O2. *Phys. Chem. Chem. Phys.* **15**, 19119-19124, doi:10.1039/c3cp52466c (2013).

12 Passananti, M. *et al.* How well can we predict cluster fragmentation inside a mass spectrometer? *Chem. Commun.* **55**, 5946-5949, doi:10.1039/c9cc02896j (2019).

13 Saiz-Lopez, A. *et al.* Iodine chemistry in the troposphere and its effect on ozone. *Atmos. Chem. Phys.* **14**, 13119-13143, doi:10.5194/acp-14-13119-2014 (2014).

14 Saiz-Lopez, A., Saunders, R. W., Joseph, D. M., Ashworth, S. H. & Plane, J. M. C. Absolute absorption cross-section and photolysis rate of I_2. *Atmos. Chem. Phys.* **4**, 1443-1450 (2004).

15 Spietz, P., Gómez Martín, J. C. & Burrows, J. P. Effects of column density on I_2 spectroscopy and a determination of I_2 absorption cross section at 500 nm. *Atmos. Chem. Phys.* **6**, 2177-2191 (2006).

16 Bogumil, K. *et al.* Measurements of molecular absorption spectra with the SCIAMACHY pre-flight model: instrument characterization and reference data for atmospheric remote-sensing in the 230–2380 nm region. *Journal of Photochemistry and Photobiology A: Chemistry* **157**, 167-184, doi:10.1016/s1010-6030(03)00062-5 (2003).

17 Gómez Martín, J. C. *et al.* On the mechanism of iodine oxide particle formation. *Phys. Chem. Chem. Phys.* **15**, 15612-15622, doi:10.1039/c3cp51217g (2013).

18 Zhang, Z. *et al.* Experimental Determination of the Ionization Energy of IO($X^2\Pi_{3/2}$) and Estimations of $\Delta_f H°_0$(IO+) and PA(IO). *J. Phys. Chem.* **100**, 63-68 (1996).

19 Wei, N. *et al.* VUV photoionization aerosol mass spectrometric study on the iodine oxide particles formed from O3-initiated photooxidation of diiodomethane (CH2I2). *RSC Advances* **7**, 56779-56787, doi:10.1039/c7ra11413c (2017).

20 Monks, P. S. *et al.* Discharge Flow-Photoionization Mass Spectrometric Study of HOI: Photoionization Efficiency Spectrum and Ionization Energy. *J. Phys. Chem.* **99**, 16566-16570, doi:10.1021/j100045a013 (1995).

21 Galvez, O., Gómez Martín, J. C., Gomez, P. C., Saiz-Lopez, A. & Pacios, L. F. A theoretical study on the formation of iodine oxide aggregates and monohydrates. *Phys. Chem. Chem. Phys.* **15**, 15572-15583, doi:10.1039/c3cp51219c (2013).

22 Rissanen, M. P., Mikkilä, J., Iyer, S. & Hakala, J. Multi-scheme chemical ionization inlet (MION) for fast switching of reagent ion chemistry in atmospheric pressure chemical ionization mass spectrometry (CIMS) applications. *Atmos. Meas. Tech.* **12**, 6635-6646, doi:10.5194/amt-12-6635-2019 (2019).

23 Caldwell, G. W., Masucci, J. A. & Ikonomou, M. G. Negative ion chemical ionization mass spectrometry—binding of molecules to bromide and iodide anions. *Org. Mass Spectrom.* **24**, 8-14, doi:10.1002/oms.1210240103 (1989).

24 Veres, P. *et al.* Development of negative-ion proton-transfer chemical-ionization mass spectrometry (NI-PT-CIMS) for the measurement of gas-phase organic acids in the atmosphere. *Int. J. Mass spectrom.* **274**, 48-55, doi:https://doi.org/10.1016/j.ijms.2008.04.032 (2008).

25 Glukhovtsev, M. N., Pross, A., McGrath, M. P. & Radom, L. Extension of Gaussian-2 (G2) theory to bromine- and iodine-containing molecules: Use of effective core potentials. *J. Chem. Phys.* **103**, 1878-1885 (1995).

26 Mahajan, A. S. *et al.* Concurrent observations of atomic iodine, molecular iodine and ultrafine particles in a coastal environment. *Atmos. Chem. Phys.* **10**, 27227-27253 (2010).

27 Saiz-Lopez, A. *et al.* On the vertical distribution of boundary layer halogens over coastal Antarctica: implications for O_3, HO_x, NO_x and the Hg lifetime. *Atmos. Chem. Phys.* **8**, 887-900 (2008).
