# Supplementary material for: A gas-to-particle conversion mechanism helps to explain atmospheric particle formation through clustering of iodine oxides
Source: Nat Commun. 2020 Sep 9;11:4521. doi: 10.1038/s41467-020-18252-8 (PMC7481236; doi:10.1038/s41467-020-18252-8)
Supplement: Supplementary file 3 — Description of Additional Supplementary Information [file 41467_2020_18252_MOESM3_ESM.pdf]

Description of Additional Supplementary Files

File Name: Supplementary Movie 1

Description: Laboratory observation of iodine smoke produced by broad band photolysis of I2 in the presence of O3, with no water added. The lamp visible light scattered by the smoke particles is observed through a view port situated the side of the flow tube.

File Name: Supplementary Movie 2

Description: Born-Oppenheimer Molecular Dynamics (BOMD) simulations. The water droplet system contained 191 water molecules and one I2O2 molecule and was simulated for 50 picoseconds.

File Name: Supplementary Movie 3

Description: As Supplementary Movie 2 for I2O3 and 80 picoseconds simulation time.

File Name: Supplementary Movie 4

Description: As Supplementary Movie 3 for I2O4 and 33 picoseconds simulation time.
